# Exploring the Role of Tripeptides in Wound Healing and Skin Regeneration: A Comprehensive Review

**DOI:** 10.7150/ijms.118118

**Published:** 2025-10-01

**Authors:** Siti Balqis Adnan, Manira Maarof, Mh Busra Fauzi, Nur Izzah Md Fadilah

**Affiliations:** 1 Department of Tissue Engineering and Regenerative Medicine (DTERM), Faculty of Medicine, Universiti Kebangsaan Malaysia, 56000 Cheras, Kuala Lumpur, Malaysia.; 2 Advance Bioactive Materials-Cells UKM Research Group, Universiti Kebangsaan Malaysia, 43600 Bangi, Selangor, Malaysia.; 3 Ageing and Degenerative Disease UKM Research Group, Universiti Kebangsaan Malaysia, 43600 Bangi, Selangor, Malaysia.; 4 Pharmaceuticals and Pharmacy Practice UKM Research Group, Universiti Kebangsaan Malaysia, 43600 Bangi, Selangor, Malaysia.

**Keywords:** Tripeptides, short peptides, wound healing, skin regeneration, therapeutic applications.

## Abstract

Wound healing is a complex and dynamic process that requires the coordination of cellular, molecular, and physiological events to restore tissue integrity. Despite notable advances in treatment strategies, optimizing healing outcomes, particularly in chronic wounds, remains a major challenge. Emerging evidence highlights the therapeutic promise of peptides, especially tripeptides, in accelerating tissue repair through diverse mechanisms. These short peptides regulate key processes such as cell migration, proliferation, and differentiation, while also modulating inflammation, promoting angiogenesis, and facilitating extracellular matrix (ECM) remodeling. This review, covering studies published between 2016 and 2025, explores the role of tripeptides in enhancing wound repair, emphasizing their biological functions, mechanisms of action, and therapeutic applications. Recent findings demonstrate that tripeptides can stimulate fibroblast migration, enhance collagen deposition, and support angiogenesis. In addition, they exhibit antimicrobial and anti-inflammatory properties, making them valuable candidates for both acute and chronic wound management. GHK-based formulations, including nanoparticle conjugates, hydrogels, and clinical derivatives such as TriHex and TriHex 2.0, enhance fibroblast migration, ECM remodeling, collagen and elastin synthesis, and wound closure while providing antimicrobial activity. KdPT mitigates hyperglycemia-induced oxidative stress and restores keratinocyte function, whereas KPV-loaded hydrogels reduce inflammation, promote tissue regeneration, and combat MRSA infections. Additionally, lipotripeptides (DICAMs) inhibit and disrupt bacterial biofilms, and GPE supports neuroprotection and regeneration through ERK and PI3K/Akt signaling activation. Beyond wound repair, this review also discusses comparative physicochemical properties and wound healing applications of tripeptides versus larger peptides, factors influencing their performance, strategies for combination with biomaterial scaffolds, and emerging applications in fields such as cancer and cosmetics. Collectively, tripeptides represent a promising class of multifunctional bioactive molecules in wound care, offering novel avenues for targeted tissue regeneration. Future research should focus on improving their stability, bioavailability, and delivery systems to fully harness their clinical potential in regenerative medicine.

## 1. Introduction

**Figure 1:**
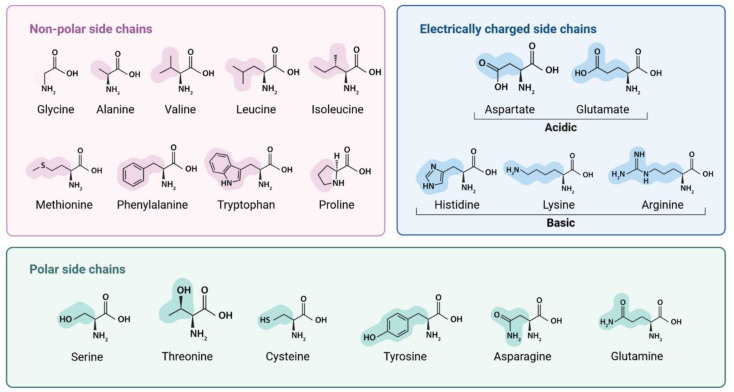
**Classification of amino acids by side chain properties: non-polar, polar, charged. Illustration created with BioRender.com**.

Therapeutic peptides are emerging as promising drug candidates, garnering significant attention in the field of medicine and drug development due to their high specificity, affinity, and low toxicity 1-3. They are being investigated for a wide range of diseases, including cancer 4, metabolic disorders 5, autoimmune conditions 6, and wound healing 7, 8. Thus, peptides became an invaluable tool in both research and medical applications. Positioned molecularly between proteins and small molecules 9, peptides typically consist of two to fifty amino acids linked by peptide (amide) bonds (-CO-NH-) 10-12 . All amino acids share a central carbon (Cα) bonded to an amino group (-NH₂), a carboxyl group (-COOH), a hydrogen atom, and a variable side chain (R group). The nature of the R group determines each amino acid's chemical properties and behavior in protein structures 13, 14. **Figure 1** illustrates this structural variability and its implications for peptide functionality. Peptides are essential signaling molecules involved in various physiological processes, including metabolism, reproduction, and immune responses. They can function as neurotransmitters, hormones, and neuromodulators in receptor-mediated pathways 15. These molecules can be derived from natural sources, including food, venoms, and marine organisms 16, 17, or synthesized through enzymatic hydrolysis and recombinant DNA technologies 18. Over the decades, extensive research has underscored the therapeutic potential of peptides in the health industry. The evolution of peptide applications, propelled by advancements in biomedical technology, has led to significant research efforts aimed at harnessing peptides for the treatment of a wide range of diseases 9.

In the context of wound healing, peptides are increasingly seen as promising agents for improving outcomes in both chronic and acute wounds due to their versatility, targeted mechanisms, and low immunogenicity. They regulate key biological processes such as inflammation, cellular migration, proliferation, and angiogenesis 19, 20. For example, basic fibroblast growth factor (bFGF) and epidermal growth factor (EGF) promote fibroblast activation, collagen synthesis, and vascularization 3, while antimicrobial peptides (AMPs) contribute to infection control and immune modulation at wound sites 21, 22. Synthetic peptides, such as P1, derived from collagen, also enhance extracellular matrix (ECM) remodeling, thereby accelerating wound repair. Despite these advantages, peptide-based therapies face significant limitations. Proteases rapidly degrade many peptides, exhibit poor oral bioavailability due to enzymatic breakdown in the digestive tract, and suffer from short systemic half-lives, reducing their clinical efficacy. These challenges have led to the development of more stable analogues, improved delivery systems, and increased interest in smaller peptide fragments that retain bioactivity while minimizing these drawbacks 9.

The emergence of tripeptides as therapeutic agents marks a significant milestone in peptide research. Early studies on Gly-His-Lys (GHK), first discovered in human plasma in the 1970s, demonstrated their ability to modulate gene expression, stimulate collagen synthesis, and accelerate wound healing, sparking broad interest in these bioactive molecules 23. Similarly, Gly-Pro-Glu (GPE) has shown neuroprotective and anti-inflammatory properties, expanding tripeptides' potential beyond dermal repair 24. Although much of the research has focused on larger peptides and growth factors 25, 26, smaller peptides, particularly tripeptides composed of only three amino acids, are now gaining attention for their efficiency. Classified as the smallest functional units within peptide categories (ultrashort: 2-9, short: 10-24, medium: 25-50, long: 50-100 amino acids) **27, 28**, tripeptides offer unique therapeutic benefits, including enhanced tissue penetration, reduced immunogenicity, and improved cellular uptake 25, 29-31.

Recent milestones in tripeptide research further underscore their therapeutic relevance. For example, topical application of GHK-Cu (a copper-bound form of GHK) has been clinically investigated for enhancing skin repair, reducing inflammation, and promoting angiogenesis. These studies laid the groundwork for exploring tripeptides not only as cosmetic agents but also as viable therapeutics for chronic wound management and regenerative medicine 24, 32-36. Given these favorable properties, further investigation into the mechanisms through which tripeptides interact with skin cells and the ECM may uncover new strategies for improving tissue repair 24, 32-36. This review aims to explore these mechanisms in greater detail, highlighting the potential of tripeptides as a novel and effective therapeutic approach for improving wound healing outcomes.

## 2. Data Extraction Management

A literature search was conducted covering studies published between 2016 and 2025 using platforms including PubMed, Web of Science (WoS), Scopus, and Google Scholar. The search strategy used the terms 'tripeptides', 'short peptides', 'wound healing', 'skin regeneration', and 'therapeutic applications'. Inclusion criteria encompassed all studies employing *in vitro* models (e.g., fibroblast proliferation, keratinocyte migration, collagen synthesis assays), *in vivo* studies using animal models for wound healing assessments, clinical trials evaluating the efficacy and safety of tripeptides in human subjects, and studies utilizing molecular, biochemical, or histological analyses to determine the mechanisms of tripeptides in wound recovery. The exclusion criteria for this review would be all secondary literature and any original articles written and submitted in languages other than English.

## 3. Tripeptide-based Therapeutics for Wound Healing and Skin Regeneration

### 3.1 Overview of Therapeutic Tripeptides Following Skin Injury

**Figure 2:**
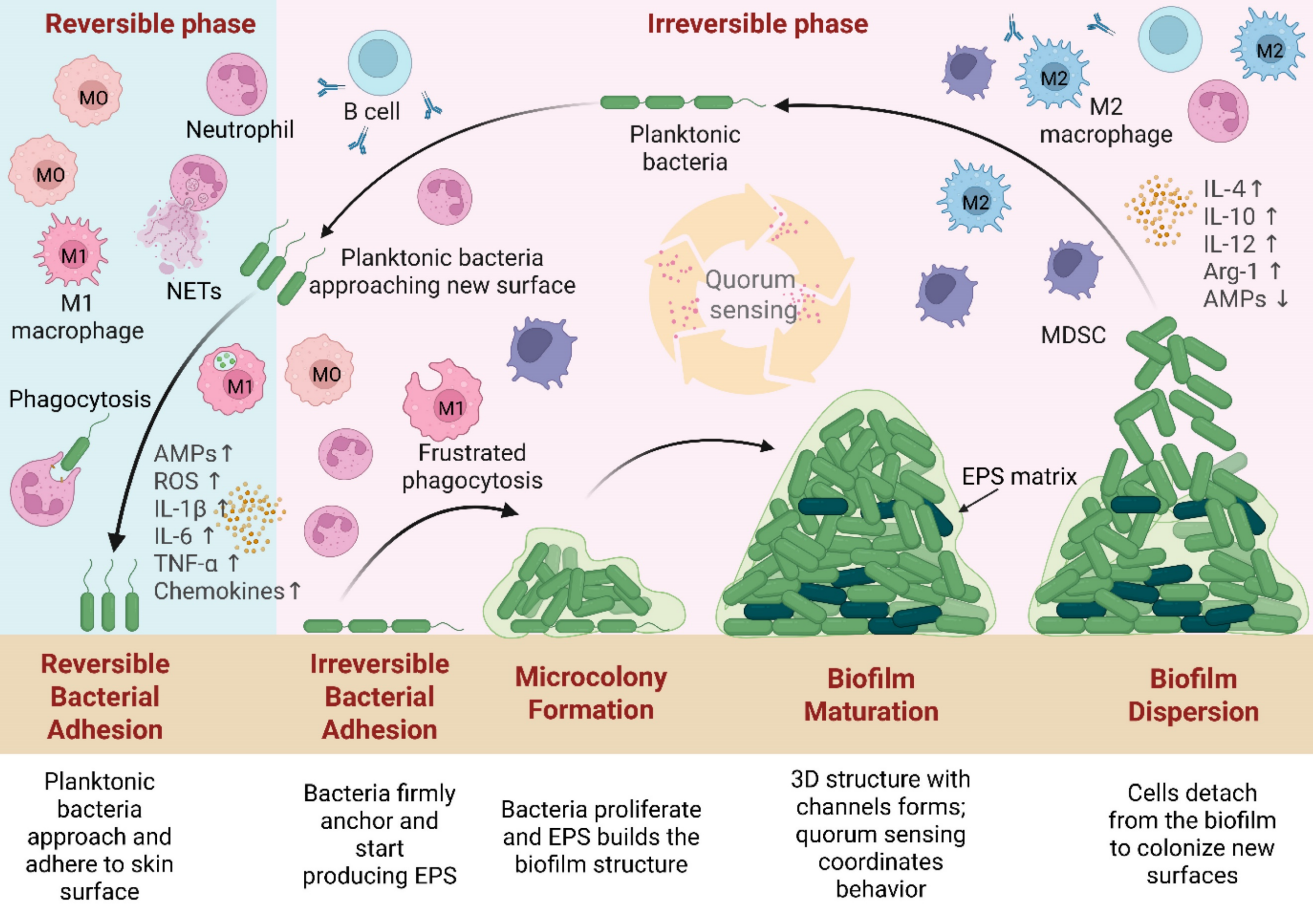
** Biofilm life cycle and associated immune responses in chronic wounds.** Following injury, planktonic bacteria approach and reversibly adhere to the skin surface. During irreversible attachment, they firmly anchor and initiate the production of extracellular polymeric substances (EPS). Microcolony formation follows, with bacterial proliferation and matrix development. During the maturation stage, the biofilm forms a complex 3D structure with nutrient and waste channels, which is sustained by continued EPS production and regulated by quorum sensing. Finally, in dispersion, bacteria are released from the biofilm and return to the planktonic state to colonize new sites. In the early adhesion phase, immune defenses are active: neutrophils and M1 macrophages target bacteria via phagocytosis, oxidative bursts, increased antimicrobial peptides (AMPs↑), reactive oxygen species (ROS↑), and pro-inflammatory cytokines (IL-1β↑, IL-6↑, TNF-α↑) and chemokines. As biofilms progress to irreversible attachment and microcolony formation, EPS reduces immune clearance efficiency. Maturation skews macrophage polarization from M1 to M2 (↑interleukin-4 [IL-4], ↑interleukin-10 [IL-10], ↑IL-12, ↑Arginase 1 [Arg-1]; AMPs↓) and activates myeloid-derived suppressor cells (MDSCs), suppressing immune function. Quorum sensing during maturation regulates EPS maintenance and enzyme release for dispersion. Illustration created with BioRender.com.

The process of wound healing is intricate and cascaded, particularly in chronic wounds like severe burns and diabetic wounds, where unfavorable endogenous or external variables can impede the healing process by interfering with the regulation of inflammation 37. More than 300 million people are impacted by wound infections each year as a result of the rising prevalence of skin injuries 38. Skin is the biggest organ of the human body, which provides maintenance of bodily fluid homeostasis and acts as a physical barrier against the entry of microorganisms 39. Following an injury, the skin bed becomes susceptible to bacterial infection, which can lead to the formation of biofilms. To better understand biofilm development and persistence in chronic wounds, **Figure 2** illustrates the stages of the biofilm life cycle, including adhesion, microcolony formation, maturation, and dispersion, as well as host immune responses such as neutrophil recruitment, M1/M2 macrophage polarization, and cytokine signaling (e.g., interleukin-1 beta [IL-1β], tumor necrosis factor-alpha [TNF-α], interleukin-6 [IL-6], interleukin-8 [IL-8], interleukin-12 [IL-12]), which collectively contribute to immune evasion and chronic infection 40-43.

**Figure 3:**
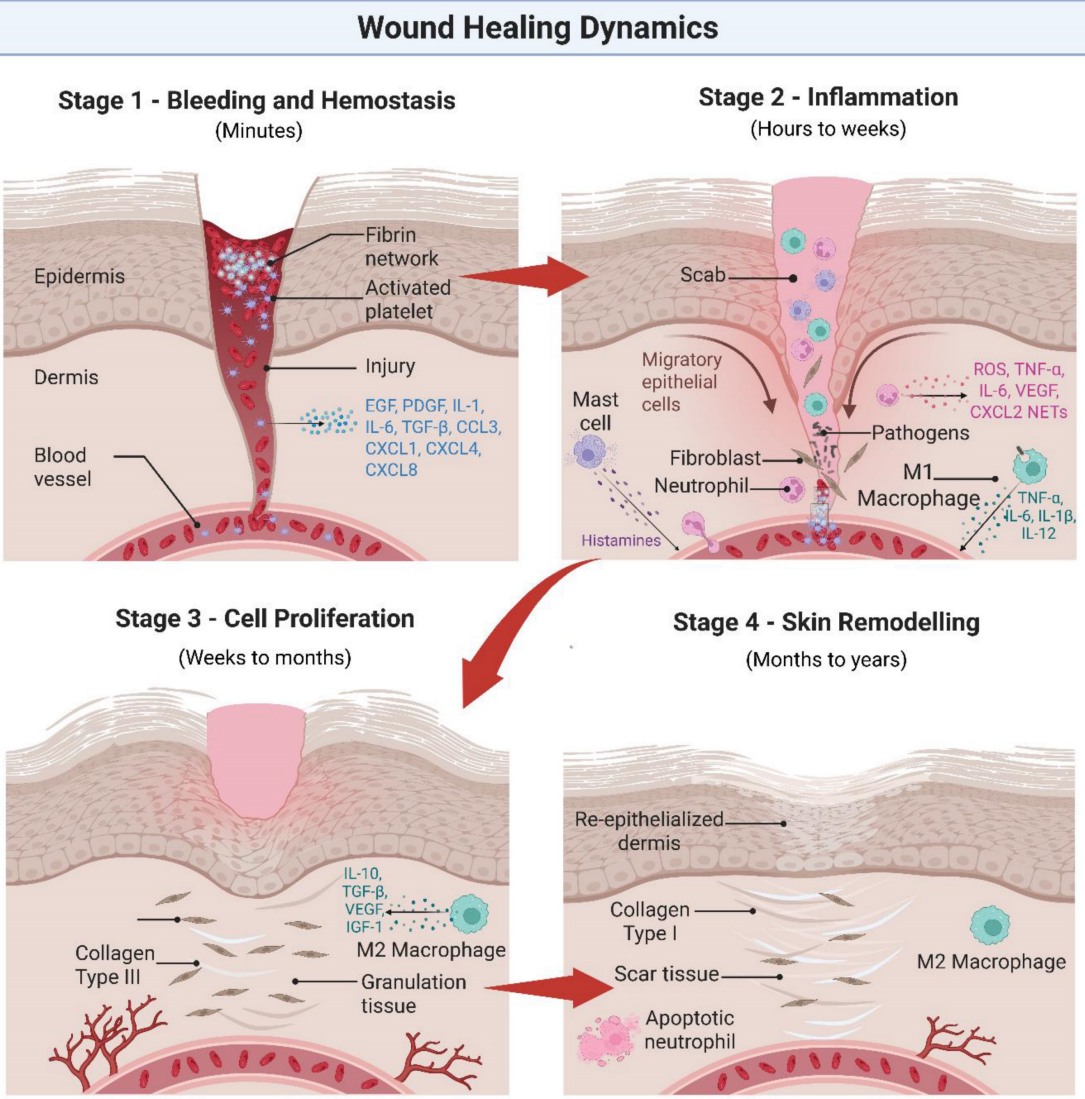
**The schematic illustrates the sequential phases of wound healing and the interplay between key cellular actors (neutrophils, macrophages, fibroblasts, keratinocytes, endothelial cells) and cytokines (e.g., TNF-α, IL-6, TGF-β, IL-10).** While normal wound healing progresses through controlled inflammation, proliferation, and remodeling, chronic wounds disrupted by bacterial biofilms experience prolonged inflammation, impaired angiogenesis, and delayed tissue restoration. Illustration created with BioRender.com.

Initially, the human body will undergo a natural healing process encompassing blood clotting, inflammation, tissue growth, and skin remodeling 44. However, bacterial biofilm in chronic wounds may cause irreversible bacterial adhesion, leading to persistent inflammation, tissue damage, and ongoing infection 43, 45, 46. Common pathogens, such as *Pseudomonas aeruginosa* (*P. aeruginosa*), *Escherichia coli* (*E. coli*), and *Staphylococcus aureus* (*S. aureus*) 47, prolong the inflammatory phase by stimulating continuous immune activation. This results in the recruitment of immune cells and the release of pro-inflammatory cytokines, which in turn influence keratinocyte and fibroblast activity 43, 47. However, biofilm-mediated disruption of granulation tissue migration interferes with re-epithelialization, angiogenesis, and skin restoration 49. **Figure 3** below summarizes these processes, highlighting the interplay between bacterial infection, immune mediators (e.g., TNF-α, IL-6, transforming growth factor-beta [TGF-β], IL-10), and cellular actors (e.g., neutrophils, M1/M2 macrophages, fibroblasts, keratinocytes, endothelial cells) during wound healing 43, 48.

**Figure 4:**
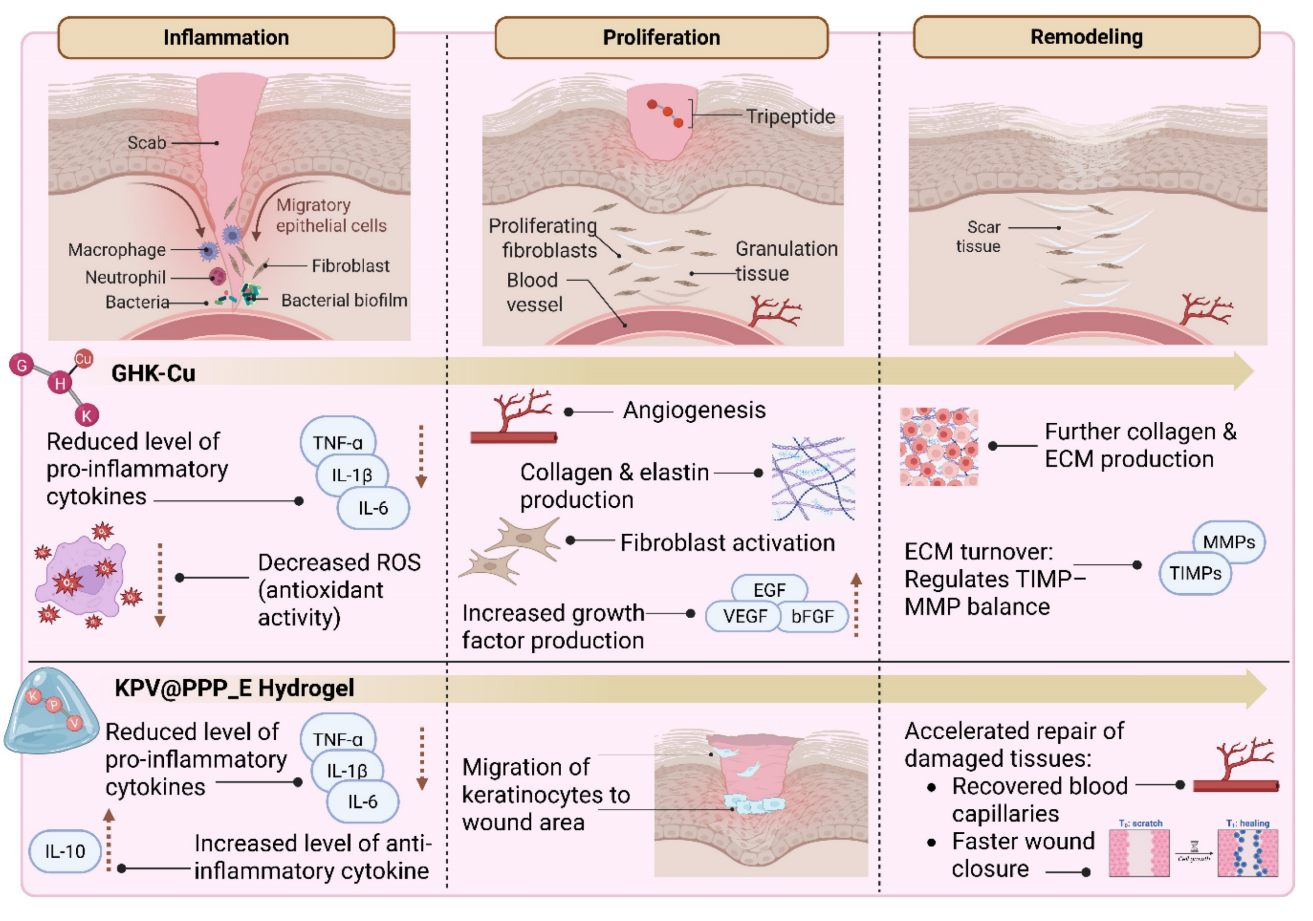
** The schematic illustrates the role of bioactive tripeptides in different stages of wound healing.** GHK-Cu promotes fibroblast proliferation, collagen synthesis, angiogenesis, and ECM remodeling, while KPV, including advanced formulations such as KPV@PPP_E, exerts anti-inflammatory effects and supports tissue repair. Together, these examples demonstrate how tripeptide-based approaches can modulate inflammation, proliferation, and remodeling to accelerate wound healing. Besides these stage-specific effects, GHK-Cu and KPV@PPP_E also demonstrated antibacterial activity, which may further support inflammation resolution by reducing microbial burden. Illustration created with BioRender.com.

In recent years, there has been a growing interest in developing targeted therapies to enhance wound healing and promote skin regeneration. Traditional approaches, such as antibiotics, wound dressings, and surgical interventions, often show limited efficacy in addressing the underlying cellular dysfunctions that impair optimal healing. This has prompted the exploration of novel molecular strategies, including the use of peptides as therapeutic agents 49-53. Among these, tripeptides, which are composed of only three amino acids, represent an emerging and promising area of biomedical research. Their cost-effectiveness and favorable pharmacokinetic properties (e.g., efficient absorption and potent bioactivity) make them attractive candidates for localized and targeted wound therapies 29, 54-57. **Figure 4** presents an overview of tripeptide-based strategies for wound healing, illustrated according to the distinct stages of tissue repair. Bioactive tripeptides such as GHK-Cu and Lysine-Proline-Valine (KPV), including advanced formulations like KPV incorporated into *in situ* mucoadhesive hydrogels (KPV@PPP_E), can influence key biological events. These include the regulation of inflammation, promotion of cell migration and fibroblast proliferation, stimulation of collagen synthesis, enhancement of angiogenesis, and remodeling of the ECM 24, 32-36, 58-60.

### 3.2 Tripeptides vs. Larger Peptides in Wound Healing Applications

While tripeptides have been introduced as promising therapeutic agents in earlier sections, this part contrasts their therapeutic potential with larger peptides, based on their physicochemical characteristics and performance in wound healing models. One of the primary advantages of tripeptides lies in their ability to interact with specific cellular receptors and signaling pathways, enabling them to influence diverse aspects of the wound healing cascade 54. For instance, some tripeptides promote fibroblast activity and collagen deposition 59, 61-64, while others enhance angiogenesis and modulate inflammation to accelerate tissue regeneration 58, 60, 65, 66. Their favorable bioactivity profile, combined with high tissue penetration and low immunogenicity, supports their integration into advanced delivery platforms such as hydrogels or topical formulations 14, 29. As shown in **Table 1**, tripeptides differ significantly from larger peptides in several biological properties. This discussion compares tripeptides, exemplified by the well-studied GHK, with larger peptides such as the cyclic heptapeptide CyRL-QN15, the human cathelicidin-derived antimicrobial peptide LL-37, and the peptide Andersonin W-1 (AW1), containing 7, 37, and 72 amino acids, respectively 25, 67, 68.

Beyond their bioactivity, tripeptides exhibit physicochemical properties that enhance their suitability for therapeutic applications. Their small, linear structures facilitate easier synthesis, high water solubility, and effective diffusion across biological barriers. Compared to larger peptides, which often struggle with poor bioavailability and proteolytic degradation, tripeptides offer a balance of functional stability and conformational flexibility. For instance, while cyclic peptides like CyRL-QN15 gain enzymatic resistance through rigid structures 84, tripeptides such as GHK maintain therapeutic potency while being more adaptable and versatile in formulation. In contrast, larger linear peptides, such as LL-37 and AW1, may exhibit strong antimicrobial activity but often face challenges related to delivery and stability. These differences highlight the intrinsic advantages of tripeptides in both drug design and localized wound healing applications 14, 29, 68, 81. While the size difference between tripeptides and heptapeptides (e.g., CyRL-QN15) may appear modest in terms of amino acid count, this difference may significantly influence their structural complexity, biological activity, and interaction with cellular targets. Therefore, more comparative studies examining the wound healing effects of GHK and larger peptides are needed to provide robust evidence on how peptide size and structural complexity influence therapeutic efficacy.

Based on the current research landscape, there is a notable scarcity of studies that directly compare the wound healing effects of tripeptides like GHK with those of larger peptides. Most existing investigations tend to focus on either small peptides or larger bioactive peptides independently, resulting in limited data on their relative efficacy, mechanistic differences, and potential synergistic applications within the same experimental frameworks. This gap underscores the need for comprehensive comparative studies to gain a deeper understanding of how peptide size and complexity impact therapeutic outcomes in wound healing. **Table 2** outlines the applications of the GHK peptide with larger bioactive peptides such as CyRL-QN15, LL-37, and AW1 across different wound healing models. This table summarizes and compares the wound healing models, key mechanisms, and therapeutic outcomes of GHK peptides and larger bioactive peptides, highlighting their shared and distinct roles to inform future research and clinical applications.

**Table 2** highlights distinct mechanisms and outcomes of peptides across wound healing models. Based on the recent studies, GHK promotes healing mainly through copper-mediated ECM synthesis and angiogenesis, supporting epithelialization and remodeling with simple delivery 23, 36, 85. CyRL-QN15 adds targeted inflammatory modulation and hair follicle regeneration via miRNA and Wnt/β-catenin pathways 86, 87. LL-37 offers strong antimicrobial and immunomodulatory effects critical for infected wounds, enhancing tissue repair and angiogenesis 88. AW1 further drives immune regulation and angiogenesis through TLR4-mediated macrophage polarization 25. These differences shed light on the impact of peptide size and complexity on therapeutic potential and guide the selection of peptides best suited for specific wound types. Combining peptides with complementary mechanisms holds promise for advancing wound healing therapies. However, comprehensive studies comparing various tripeptides and larger peptides are needed to validate these distinctions and fully elucidate their effects on wound healing efficacy.

### 3.3 Examples of Specific Tripeptides with Proven Efficacy

While the comparative advantages of tripeptides over larger peptides highlight their therapeutic potential, it is through specific examples that their true value becomes evident. Several tripeptides have been extensively studied for their roles in modulating wound healing pathways, offering concrete evidence of their efficacy in both preclinical and clinical contexts. Through both *in vitro* and *in vivo* studies, a variety of tripeptides have been identified as effective in enhancing wound healing and skin regeneration. These peptides act on different stages of the healing process, such as reducing oxidative stress, enhancing angiogenesis, and accelerating epithelialization. By targeting specific biological pathways, tripeptides not only accelerate recovery but also help to improve the quality of the healed tissue, resulting in more resilient and youthful-looking skin 24, 36, 58, 61, 85, 89-91. The following **Table 3** outlines several tripeptides that have been the subject of research into their efficacy in wound healing and skin regeneration, providing further insight into their diverse mechanisms of action. Each tripeptide has shown promise in experimental settings, making them valuable candidates for future therapeutic applications in dermatology and regenerative medicine.

#### 3.3.1 GHK-AgNPs and GHK-CuNPs

The wound-healing potential of novel tripeptides GHK-AgNPs and GHK-Cu-AgNPs was assessed both *in vitro* using mouse dermal fibroblasts (PAM212 and L929) and *in vivo* in *S. aureus*-infected mouse models. Both treatments enhanced fibroblast migration and recovery. In L929 cells, wound closure after 12 hours reached 83% with GHK-AgNPs and 76% with GHK-Cu-AgNPs; by 24 hours, closure increased to 96% and 92%, respectively. In PAM212 cells, 12-hour closure was lower but still notable, achieving 87% (GHK-AgNPs) and 90% (GHK-Cu-AgNPs) at 24 hours, both significantly higher than controls. On Day 3 post-injury, GHK-AgNPs and GHK-Cu-AgNPs achieved the highest closure rates (32% and 31%, respectively) compared with GHK-Cu and the positive control (both 25%) and the negative control (22%). By Day 7, GHK-AgNPs maintained the fastest healing rate, followed by GHK-Cu-AgNPs, the positive control, GHK-Cu, and the untreated control. Significant acceleration in wound repair was evident on Days 7 and 11 (*p*< 0.01). By Day 11, closure reached 96% for GHK-AgNPs and 94% for GHK-Cu-AgNPs. Histological analysis confirmed these findings, showing improved tissue regeneration, increased epidermal thickness, greater collagen deposition, and reduced TNF-α expression, collectively indicating a more efficient healing process. These results suggest that conjugating GHK with silver or copper nanoparticles substantially enhances its wound-healing efficacy, potentially offering a powerful therapeutic approach for infected wounds 36 (**Table 3**).

However, several limitations remain. These include the short *in vivo* half-life of tripeptides (approximately <30 min in plasma) 92, instability of metal-peptide complexes (particularly GHK-Cu, owing to copper's high reactivity and sensitivity to environmental factors), poor skin absorption due to high hydrophilicity, and rapid clearance after dermal injection, with about 95% excreted 93. In addition, potential cytotoxicity from metal nanoparticle conjugates (e.g., nano silver-induced toxicity) 36, 94 and the lack of validation in chronic wound models using medium- to large-sized experimental animals, along with limited human clinical trials, remains a significant barrier to the clinical translation of GHK 75, 95.

Nanoparticle-based delivery systems offer a promising strategy to address these limitations by improving peptide stability, prolonging systemic circulation, enhancing tissue penetration, and reducing off-target effects. Tripeptide conjugation potentially enhances nanoparticle stability, modulates release kinetics, and imparts bioactivity. By modifying surface charge, providing steric protection, and forming strong coordination bonds, conjugated peptides reduce aggregation, resist degradation, and maintain integrity under varying pH. Evidence from anthocyanin nanoencapsulation studies shows that tripeptides (e.g., LWD, LWE, LWH) can form stable nanocomplexes through π-π stacking, hydrogen bonding, and hydrophobic interactions, markedly improving stability against heat, pH fluctuations, and metal ion stress. Such interactions not only preserve bioactive compounds but also suggest that tuning peptide composition can further optimize nanocarrier performance 36, 96, 97.

Additionally, bioactive tripeptides potentially improve targeting, promote self-assembly, and adjust surface hydrophilicity. Ultimately, the performance of peptide-nanoparticle conjugates (PNCs) depends on design parameters such as size, shape, surface charge, peptide choice, and conjugation chemistry, which determine their precision and capacity to overcome biological and physical barriers. A deeper understanding of these properties can guide the design of PNCs with enhanced specificity and responsiveness, enabling more effective targeted delivery and precise therapeutic action in wound healing applications 36, 96, 97.

#### 3.3.2 KdPT

While GHK-based formulations have shown strong potential in infected wound models, other tripeptides have demonstrated promise in addressing wound-healing impairments arising from metabolic disorders such as diabetes. The chronic hyperglycemic environment in diabetic patients disrupts the normal process of wound healing. Chronic foot ulcers are a major complication in patients with diabetes mellitus, often leading to significant morbidity. Hyperglycemia, commonly observed in diabetic patients, impairs keratinocyte functions involved in wound re-epithelialization, affecting the complete restoration of the epidermal skin barrier through cell migration and proliferation. Although the precise mechanisms remain inadequately understood, chronic hyperglycemia is known to induce glucotoxicity, which is characterized by the deterioration of β-cell function due to elevated blood glucose levels. This impairs the β-cell response to glucose and increases insulin resistance 98. Glucotoxicity, which is linked to the generation of intracellular ROS, contributes to the development of foot ulcers and delayed wound healing 99.

Gkogkolou et al. 90 described that the α-MSH-derived tripeptide Lys-d-Pro-Thr (KdPT) ameliorated glucotoxicity by significantly reducing HG-mediated ROS production in NHKs in a dose-dependent manner. KdPT also counteracted the negative effects of hyperglycemic conditions on the viability, proliferation, migration, and metabolic activity of NHKs. In scratch assays, KdPT reduced the inhibition of NHK migration induced by HG, as evidenced by a decrease in the open wound area. Furthermore, in wounded human skin organ cultures, KdPT significantly attenuated the HG-mediated suppression of re-epithelialization 90 (**Table 3**). Nevertheless, clinical translation of KdPT faces several limitations. Most research on KdPT has been performed *in vitro* (e.g., keratinocytes, sebocytes) or in ex vivo models such as human skin organ cultures, as well as in inflammatory or intestinal disease models. While these findings are promising, KdPT has not yet been thoroughly evaluated in standard animal wound-healing models, particularly in chronic or diabetic contexts, which limits its translational relevance 90, 100.

### 3.3.3 Tripeptides in Wound Healing: Promise and Challenges

Tripeptides have shown significant potential in enhancing wound healing, both in normal and hyperglycemic conditions, by targeting various cellular processes involved in tissue repair. Tripeptides play a significant role in wound healing by promoting skin and tissue regeneration, stimulating angiogenesis, and accelerating collagen synthesis, which is essential for restoring skin structure. They act through interactions with specific cellular receptors, triggering key processes such as cell migration, proliferation, and differentiation required for effective wound closure. In hyperglycemic wounds, where healing is often delayed due to chronic inflammation, impaired circulation, and cellular dysfunction, tripeptides help mitigate these challenges. They modulate inflammation, reduce oxidative stress, and enhance the function of fibroblasts and keratinocytes, which are vital for tissue repair. Additionally, tripeptides support the remodeling phase by improving collagen deposition and organization, contributing to tissue strength and elasticity. Their capacity to expedite epithelialization, minimize scarring, and prevent infections makes them particularly valuable for chronic wounds, including those associated with diabetes. Overall, tripeptides offer a dual therapeutic advantage by supporting both normal and impaired wound healing processes 24, 36, 58, 61, 85, 89-91.

Despite their promise, the clinical translation of tripeptides faces several challenges, particularly related to their stability, bioavailability, and functional performance in complex wound environments. While certain di- and tripeptides have demonstrated the ability to cross intestinal membranes intact via peptide transporter systems, many remain susceptible to rapid degradation by proteases. These limitations can hinder their sustained activity at target sites. However, advancements in peptide engineering and delivery strategies, including chemical modifications, encapsulation, and conjugation, are progressively improving the ADME (absorption, distribution, metabolism, and excretion) features of peptide drugs. Coupled with an expanding body of preclinical and clinical evidence, these innovations suggest that tripeptides are well-positioned to become integral components of next-generation wound therapies 14, 29, 77. Continued research into their mechanisms of action is expected to further enhance their therapeutic applicability in treating chronic wounds, burns, and other skin disorders. Understanding the variables that influence tripeptide stability, bioavailability, and biological interactions is crucial for optimizing their clinical outcomes.

### 3.4 Factors Influencing the Performance of Tripeptides

#### 3.4.1 Stability

Tripeptides may degrade over time or in certain environments. The formulation's pH, temperature, and storage conditions can affect peptide integrity, making it crucial to use stabilizing agents or specific delivery systems to preserve their potency. Stability studies of drugs are essential for verifying the performance of tripeptide medications, particularly regarding their safety and efficacy. Drug stability is defined as the ability of a drug substance or product to retain its properties and characteristics within specified limits throughout its shelf life and under the conditions of defined storage and use 101. This will aid in determining a drug's shelf life, thus guiding medicinal drug development. Each drug ingredient can influence the stability of the drug substance and/ or dosage forms.

In general, the chemical, physical, microbiological, therapeutic, and toxicological properties of a formulation must remain within specified limits throughout its storage in the same container. In terms of chemical stability, the integrity and potency of each ingredient must also be preserved within defined limits. Meanwhile, physical characteristics should be preserved for physical stability, including appearance, uniformity, solubility, suspendability, and palatability. Resistance or sterility against microbial growth, consistent therapeutic effects, and an insignificant rise in toxicity are also required to ensure the stability of drugs. Some of the key environmental factors affecting drug stability are light, humidity, oxygen, carbon dioxide, and temperature. Besides, the main dosage form factors that can affect stability include pH, compatibility of anions and cations, solution ionic strength, particle size, molecular binding, diffusion of drugs and excipients, solvent system composition, primary container, and specific chemical additives 101, 102.

The stability of medicinal drugs can also be enhanced using dispersed systems such as liposomes, emulsions, and suspensions. Electrostatic repulsions and attractions between particles can have a major impact on these systems' stability 103. Due to the inherent limits of amino acids, peptides frequently suffer from membrane impermeability and low stability *in vivo* 15. Therefore, the incorporation of tripeptides with carrier systems such as liposomes potentially aids the stability of the system, thus optimizing the delivery of tripeptide drugs. Liposomes were employed as carriers for GHK-Cu in a study by Dymek et al. 69. The stability of the tripeptide-loaded carriers can also influence the performance of tripeptide drugs. Several evaluations of the stability of liposomes were conducted to determine the system's stability and absence of particle aggregation, including the monitoring of size and zeta potential to prove the dispersion's stability (optimal value of ± 30 mV), which determines peptide encapsulation efficiency in liposomes. The study highlighted that higher lipid composition, both quality and quantity, can enhance the stability of liposomes as carriers, which in turn influences the stability of the tripeptide drug performance. Other parameters, including pH and temperature, were kept unchanged 69. However, while the study reports encapsulation efficiency as a measure of liposomal stability, it does not provide *in vivo* pharmacokinetic data such as bioavailability, biodistribution, half-life, or clearance rates in animal models, which can significantly complement *in vitro* efficacy in identifying new drug leads. The absence of such data limits the ability to predict clinical performance and poses challenges for therapeutic translation 69, 104.

#### 3.4.2 Specificity

A tripeptide's capacity to bind specific receptors or cells dictates its selectivity, reducing off-target effects while enhancing therapeutic efficacy. Due to peptides' high specificity and activity, research on antiviral peptides has gained widespread attention. Antiviral peptides (AVP) have been shown to inhibit infection by specifically binding to the virus or the host, thus preventing viral fusion with the host cell 105. Enfuvirtide, which is the first AVP to be approved, has been used in combination therapy to treat human immunodeficiency virus (HIV) infection in adults and children since 2003. Enfuvirtide blocks HIV infection by specifically binding to the HIV envelope protein, warding off viral-host fusion 15, 106. The AVP drugs telaprevir and boceprevir were clinically approved in 2011 for the therapy of hepatitis C virus (HCV), in which they work by specifically targeting the HCV NS3/4A serine protease. As a result, protease activity will be inhibited, preventing HCV replication in the host 15, 107. The development of peptide vaccines against SARS-CoV-2 received particular focus during the COVID-19 pandemic due to their high specificity, easier synthesis, and good safety profiles. Several attempts were made to design novel COVID-19 peptide vaccine candidates, yet there are currently no approved COVID-19 peptide vaccines. Nonetheless, these efforts certainly added new insights and knowledge on peptide vaccine development that can potentially protect against both SARS-CoV-2 and emerging viruses in the future. The unique biochemical characteristics of tripeptides should be extensively leveraged to develop effective tripeptide drug candidates that can be successfully transitioned into the commercial market. Hundreds of therapeutic peptides are undergoing preclinical research and clinical development, while over 80 have already made it to the worldwide market 15.

Targeted cancer therapies are made possible by the great specificity of peptides in oncology, which offers alternatives to conventional cancer treatments like surgery and radiation therapy, which are only partially effective in advanced cancers. Peptide applications in cancer treatment fall into a few primary categories 15. Peptide-based imaging probes, such as ^177^Lutetium-[DOTA°, Tyr^3^]octreotate (^177^Lu-DOTATATE), a radiolabeled somatostatin analogue, have been found useful in cancer therapy. ^177^Lu-DOTATATE was recently approved for the treatment of gastroenteropancreatic neuroendocrine tumors (GEP-NETs) expressing somatostatin receptors (SSTRs). These receptors are overexpressed on GEP-NET cells, enabling selective binding of the drug and localized delivery of radiation. Upon receptor binding, dotatate releases lutetium-177 (^177^Lu), generating intracellular free radicals that induce cellular damage 108, 109. Further, peptides are attractive strategies for cancer treatment due to their binding to specific receptors, such as the receptors in the programmed cell death protein 1/programmed death-ligand 1 (PD-1/PD-L1) signaling pathway. In a 3D co-culture model observed by Boohaker et al. 110, the PD-L1 peptide mimic, namely PL120131, specifically bound to the PD-1, thus disrupting the interactions of PD-1/PD-L1, resulting in greater survival and activities of co-cultured T cells compared to the PD-1 antibody 110. Animal-derived toxic peptides (VPs) may also have anti-tumor properties. VPs exhibit a high degree of specificity and selectivity for particular ion channels and cell membrane receptors because they naturally target mammalian receptors 15. A peptide poison called hanatoxin-1, which was extracted from Chilean spiders, selectively inhibits the membrane's K^+^ channel 111. The development of colon cancer has been linked to elevated K^+^ channel expression. In another study, Okada et al. 112 discovered that the lung cancer cell lines LX22 and BEN, which showed an endogenous K^+^ current, were cytotoxically affected by the porogenic peptide LaFr26 that was isolated from the venom of *Lachesana* sp.

#### 3.4.3 Toxicity

Drug toxicity is a significant challenge in drug development, as it can lead to late-stage drug attrition and, thus, failure of promising drug candidates. Off-target effect, which is one of the key issues in drug toxicity, occurs as a result of the drug binding to unintended binding sites, leading to potential adverse effects. Therefore, evaluations of interactions of drugs with the biological environments are crucial to predict and mitigate toxicity risks, while accurately establishing the safety profile of the drugs 113, 114. In particular, tripeptides serve as targets for many chemicals and are involved in various biochemical reactions in the human body. Hence, toxicity evaluations of candidate tripeptide drugs are imperative. Peptides are typically utilized in cosmetic products in concentrations of less than 10 ppm, with values ranging from 1 ppm to 30 ppm.

While conjugation of AgNPs with tripeptides like GHK can improve biocompatibility, the conjugation process can sometimes introduce risks of toxicity, particularly when high concentrations of nanoparticles are used. It is important to conduct thorough biocompatibility assessments to ensure that the conjugated nanoparticles do not cause cytotoxicity or immune reactions 36. Toxicology evaluations by Johnson et al. 115 revealed that the GHK peptide is safe for cosmetic use based on the study results. The available evidence supports the safe use of these components in cosmetic products due to their low use concentrations (maximum use concentration = 0.002%), negative repeated dosage toxicity, skin irritation and sensitization, and genotoxicity data. Li et al. 74 highlighted that the immobilized Cu^2+^ and its effects in stimulating cell proliferation do not induce cytotoxicity to the primary human cells due to the complexation of copper with GHK. Dunn et al. 116 assessed the subchronic oral toxicity of the specific tripeptide arginine, alanine, and lysine (RAK), which is a potential therapeutic tripeptide for human dietary use. In the study, rat models were used in a 90-day repeated-dose toxicity of gavage RAK at dosages of 0, 250, 500, or 1000 mg/kg bw/day. No deaths or treatment-related adverse effects were observed. The maximum dosage studied, 1000 mg/kg bw/day, was shown to have a no-observed-adverse-effect level (NOAEL), further indicating the low toxicity of RAK. Another example of a tripeptide with low toxicity is the ACE-inhibitory tripeptide EWL, in which EWL at different concentrations (mM) exhibited relatively low cytotoxicity towards HepG2 cells, a human hepatoblastoma cell line, compared with the control 117. Similarly, cytotoxicity was evaluated for the tripeptides Cys-Val-Leu (CVL), Cys-Ser-Phe (CSF), and Cys-Ser-Asn (CSN), which are novel tyrosinase inhibitors. None of them showed toxicity to the reconstructed human epidermis model, suggesting their potential use as ingredients for cosmeceutical products 69. Collectively, these studies suggest comparable low toxicity risks of several potent tripeptides, indicating a higher probability of achieving great safety profiles.

#### 3.4.4 Long-Term Safety

Since wound healing treatments may require prolonged application (especially for chronic wounds like diabetic ulcers), long-term safety studies are necessary to assess any potential accumulation of nanoparticles or peptides in the body and ensure the absence of adverse effects over time. For a drug candidate to be commercialized, the drug candidate must be approved by the Food and Drug Administration (FDA) based on particular safety and efficacy standards. Recurrent analyses of drug-drug interactions, reactive metabolites, nonspecific targets, cardiotoxicity, mutagenicity, cytotoxicity, and teratogenicity are incorporated to assess the safety of a molecule 114. Anti-inflammatory tripeptide cream (ATPC) is a topical formulation consisting of anti-inflammatory tripeptides, Binterin and Winhibin. Yang et al. 66 revealed that the long-term (9 weeks) consumption of the cream significantly alleviated grade ≥ 2 hand-foot syndrome (HFS) and/ or hand-foot skin reaction (HFSR) development in patients with existing HFS/HFSR in comparison to the group receiving the placebo cream. HFS/HFSR are common adverse events caused by molecular-targeted multi-kinase inhibitors (MKIs) or cytotoxic chemotherapy agents that can affect a patient's quality of life (QoL), thus influencing physicians' decisions to either reduce treatment dosage or discontinue the anticancer drugs, which consequently will reduce the efficacy of given cancer treatments. In contrast, conventional medications include celecoxib (long-term adverse effects on the upper gastrointestinal tract or heart), urea cream (not containing anti-inflammatory properties), and topical corticosteroids (weeks to months of consumption may induce local adverse effects, like pustular psoriasis, large capillaries, easy bruising, or weakened skin) 66. Therefore, this study proposes the long-term safety of anti-inflammatory tripeptides Binterin and Winhibin.

The long-term safety of the transdermal drug delivery system of curcumin liposomes with skin-penetrating peptides (TD-1) is considered safer than traditional delivery methods such as intravenous injection and oral, making it favorable for long-term disease treatments, such as cancer 118. Curcumin, which has antitumor, anti-inflammatory, and antioxidant properties, is widely known for its efficacy in treating melanoma. Intradermal application of curcumin will minimize its side effects and toxicities, providing optimal long-term safety. Certain chemical penetration enhancers are known to trigger skin irritation 119, 120, while physical penetration-promoting methods can likely cause skin damage 119. Therefore, non-invasive biological penetration enhancement procedures should be introduced 118. The methodologies include biocompatible preparations, such as liposomes, and bioactive penetration-promoting components, such as tripeptides 121, 122.

#### 3.4.5 Penetration

The capacity of the tripeptide to penetrate the skin or cellular membranes is critical for its efficacy in topical or systemic applications. This is influenced by its size, charge, and lipophilicity. Some topical pharmaceutical and cosmeceutical drugs do not require skin penetration and can elicit their effects on the surface of the skin, whereas some topical medications require transdermal penetration of bioactive ingredients for the drugs to be more efficacious. For the latter, the ability of the drugs to penetrate the skin's stratum corneum to the target site and to reduce the skin's barrier performance are prerequisites to ensure optimal performance of the drugs 123-125. The assay methodology of Franz diffusion cells is primarily used to assess the *in vitro* drug release and ex vivo skin penetration of various topical applications, including gels, creams, microemulsions, transdermal patches, and creams 105, 124-126.

Tripeptide drugs, which aim to provide therapeutic/ cosmeceutical benefits to the skin, i.e., wound healing, skin regeneration, and anti-aging, are closely associated with the challenges of permeating the skin barriers. Dymek et al. 69 demonstrated the skin penetration ability of GHK-Cu tripeptide, which can be used in patch technology to treat skin inflammations. As a complexing agent for copper ions, GHK peptide raises Cu^2+^ penetration rates, which is likely to speed up copper ion migration across the lipophilic stratum corneum 69. Park et al. 127 assessed the permeability of GHK and its derivatives (GHK-Cu and Pal-GHK), such that Pal-GHK exerted the highest percentage of permeated peptides (4.61%), followed by GHK-Cu (3.86%) and GHK (2.53%), suggesting metal complexation as a novel strategy for skin penetration enhancement. In a preliminary human skin ex vivo penetration study by Lee et al. 105 the Franz diffusion cells analysis recorded the permeability of hydrolyzed collagen tripeptide (CTP) (Gly-Pro-Hyp tripeptide) up to 6.74%. Additionally, the covalent attachment of arginine oligomers (tetra and hexa-D-arginine, R4 and R6, respectively) to the tripeptides positively contributed to skin permeability. Significantly, studies on the skin penetration abilities of distinct drug formulations are crucial to ensure excellent delivery of topical tripeptide drugs.

## 4. Combination Strategies with Biomaterial Scaffolds

**Figure 5:**
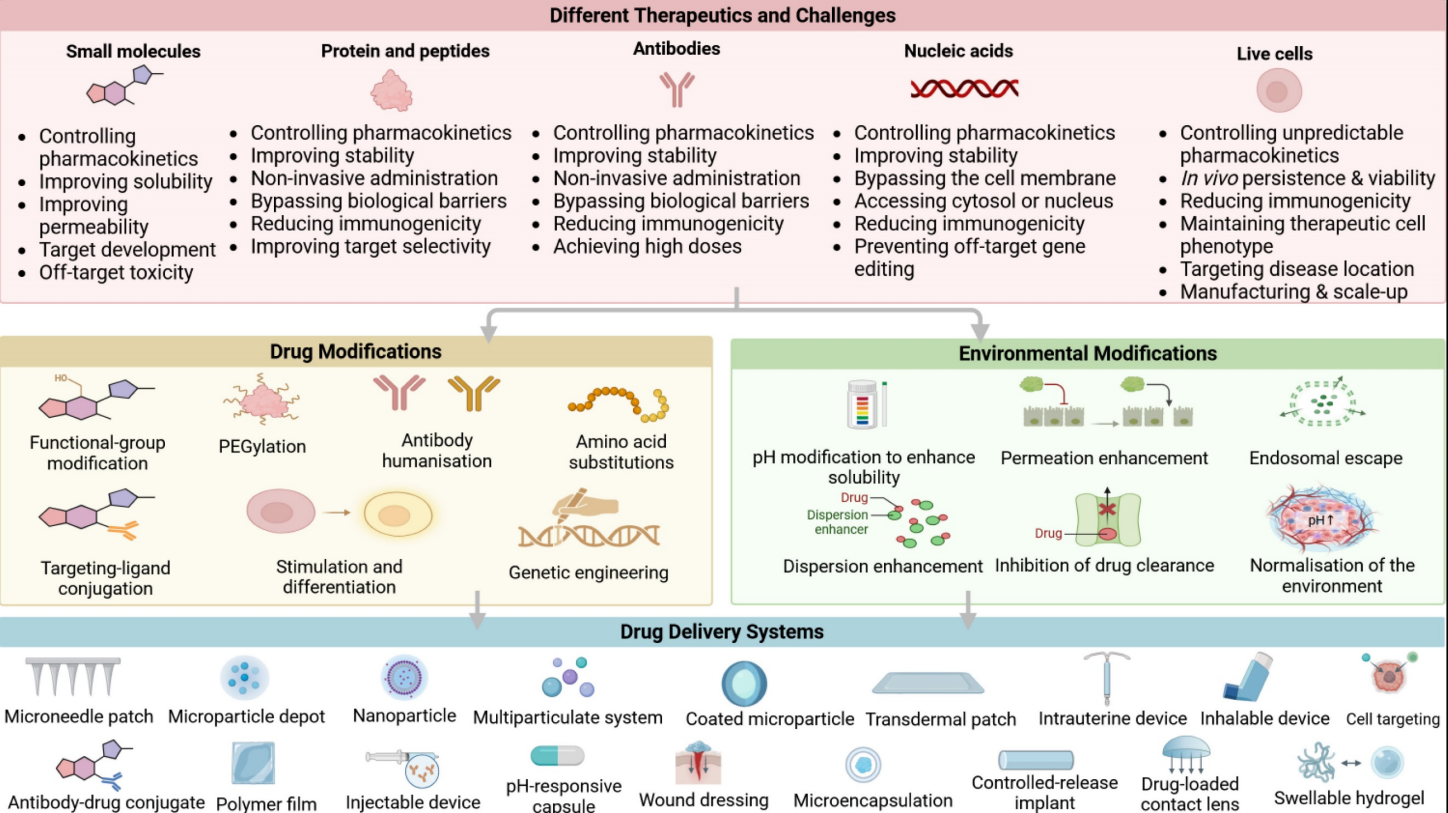
** Overview of therapeutic modalities, challenges, and advanced drug delivery strategies.** This figure illustrates the classes of therapeutics and their associated challenges, including small molecules, proteins and peptides, antibodies, nucleic acids, and live cells. Illustration created with BioRender.com.

Recent studies also demonstrated the synergistic potential of peptides in combination with other regenerative scaffolds. The integration of peptides in biomaterials opens up a variety of applications, including tissue engineering 2, 128, drug delivery systems 129, biocatalysis 130, and wound healing 85, 131. These small, peptide-based structures can spontaneously organize into well-defined nanostructures, such as hydrogels or fibrillar networks, under specific conditions. **Figure 5** shows the overview of different therapeutic modalities, challenges, and advanced drug delivery strategies.

Furthermore, the ease of synthesis, low toxicity, and the potential for large-scale production make these peptides highly attractive as candidates for clinical applications in regenerative medicine. Studies have shown that β-tripeptides can self-assemble into various structures, including nanofibers and mesh-like scaffolds, even when incorporating functional side chains or bioactive molecules 132. These scaffolds have demonstrated biocompatibility and the ability to support cell growth. The incorporation of cell adhesion motifs, such as Arg-Gly-Asp (RGD) and Ile-Lys-Val-Ala-Val (IKVAV), into peptide sequences has been explored as a strategy to enhance cell attachment and promote growth 133. However, careful tuning of bioactive peptide concentration may be necessary to avoid over-stimulation and optimize cell growth. These findings suggest that self-assembled tripeptides hold promise as versatile and biocompatible scaffolds for various tissue engineering applications.

### 4.1 Incorporation of Peptide with Hydrogels

Drug carriers such as hydrogels, liposomes, nanocarriers, and microneedles can act as vehicles when conjugated with drug-loaded molecules 134, 135. These drug delivery systems potentially enhance drug delivery to the target sites and maximize the efficacy of the drugs 136-138. Hydrogels are three-dimensional, cross-linked networks of hydrophilic polymers capable of absorbing and retaining large amounts of water or biological fluids while maintaining structural integrity 139, 140. This renders it promising for wound healing therapies, as the rationalization of hydrogels as polymers for wound dressings is supported by the concept of optimal wound healing, which requires a well-maintained moist environment essential for oxygen permeation and removal of wounds 141. The inflammatory properties of hydrogel dressings are beneficial in regulating inflammation and accelerating wound recovery through the mechanisms of free radical scavenging, stimulation of M_1_-to-M_2_ polarization of macrophages, and chemokine sequestering 37.

Peptide-incorporated hydrogels have been investigated for potential applications in wound healing. Incorporation of GHK and amino acid α-L-arginine into polysaccharide hydrogel dressings based on low-methoxyl amidated (LMA) citrus pectin or flaxseed gum was shown to accelerate wound recovery in rats compared to untreated controls. While the study did not report quantitative data on peptide loading efficiency, it provided detailed release profiles. The LMA pectin hydrogel released the tripeptide very slowly due to strong binding interactions with its carboxylic groups, which may also enhance peptide stability within the matrix. In contrast, the flaxseed gum hydrogel exhibited zero-order release kinetics, offering a constant and controlled release profile that is optimal for drug delivery. Histological analysis further revealed that complete wound healing was achieved only when the tripeptide was delivered via the flaxseed gum hydrogel. These findings highlight that the nature of tripeptide-hydrogel interactions critically influences both peptide stability and delivery efficiency, ultimately shaping therapeutic outcomes in wound healing applications 85.

KPV tripeptide, incorporated into *in situ* mucoadhesive hydrogels (KPV@PPP_E, a KPV-loaded formulation described earlier), restored the tissue morphology of ulcerated gingival mucosa in rats with chemotherapy-induced oral mucositis, partly through the upregulation of the anti-inflammatory cytokine IL-10. Contrastingly, the inflammatory cytokines such as IL-1β and TNF-α were inhibited 58. Furthermore, encapsulation of tripeptides in hydrogels allows antibacterial activities, which, in turn, enhances wound healing processes. KPV@PPP_E demonstrated antibacterial properties in gingival ulcer wounds infected with MRSA, which inhibited inflammatory cell penetration into submucosal tissues. In addition, the hydrogel system incorporated silver nanoparticles (AgNPs) with terminally 2-(naphthalene-6-yl) acetic acid-protected Phe-Phe-Cys-peptide (Nap-FFC) demonstrated antibacterial activities against both Gram-positive and Gram-negative bacteria, which were MRSA and *Acinetobacter baumannii*, respectively 142.

### 4.2 Incorporation of Peptides with Liposome

Liposomes, which are amphipathic, spherical lipid vesicles (particle size spanning about 50- 500 nm in diameter), are constituted of one or more lipid bilayers, where the hydrophilic head groups orient towards the external aqueous environment while the hydrocarbon chains aggregate within the hydrophobic interior 143, 144. Used to encapsulate and deliver drugs to the target sites, liposomes are among the most extensively studied nanocarriers for drug delivery applications and are widely used as therapeutic nanoparticles due to their biocompatibility, high bioavailability, stability, high efficiency of drug loading, and ease of synthesis 144. The amphiphilic characteristic of liposomes renders them ideal drug carriers, especially for drug molecules of different polarities 143.

The cosmetical benefits of liposomal-encapsulated tripeptides were described in several studies. Dymek et al. 69 reported that liposomes can be utilized as carriers for the copper-binding tripeptide GHK-Cu, in which GHK-Cu remarkably inhibits elastase, which lowers the rate of skin elastin degradation and regulates structural integrity, contributing to anti-aging effects in skin. GHK-Cu is a moderately hydrophilic compound with limited permeation into the stratum corneum of the skin, which is lipophilic. Liposomal encapsulation can increase GHK-Cu's ability to penetrate epidermal barriers, but there are currently insufficient and sparse studies regarding the mechanisms 145. A comprehensive study by Han et al. 63 recorded significant results elicited by the topical delivery of nanoliposomes loaded with bioactive peptides, including carnosine, palmitoyl tripeptide-5, and acetyl hexapeptide-3 (CPA-NLPs), in demonstrating skin anti-aging effects. The encapsulation endowed high encapsulation efficiency, sustained release of peptides, and high loading capacity. Compared to the control group (free CPAs), these factors greatly influenced the significant increase in cellular uptake of the loaded cargos of bioactive peptides into the human skin fibroblasts (HSF), enhanced protective effects of oxidation-damaged cells, elevated levels of the major constituents of the ECM, type Ι collagen (Col I) and hyaluronic acid (HA), reduced skin wrinkles, and increased skin elasticity. Additionally, liposomes, which potentially enhanced drug penetration through the stratum corneum, resulted in longer-lasting time of drug release to their target sites and drug efficacy 63. The combination of the bioactive peptides confers synergistic effects, as carnosine, which is an endogenous dipeptide, exhibits anti-oxidation, anti-inflammatory activities, and stimulation of collagen synthesis. Meanwhile, acetyl hexapeptide-3 is known for its anti-aging properties 63, 64.

### 4.3 Incorporation of Peptide with Nanoparticles

The incorporation of peptides with nanoparticles represents a rapidly advancing area of research in nanomedicine, biomaterials, and drug delivery systems 146. Peptides, due to their bioactivity, specificity, and biocompatibility, are increasingly recognized as promising candidates for a variety of therapeutic applications. However, their inherent limitations, such as instability, rapid degradation *in vivo*, and poor bioavailability, have prompted researchers to explore innovative methods of optimizing and improving their properties 147. Nanoparticles, owing to their high surface area, tunable size, and versatile surface chemistry, offer a compelling solution to these challenges. By conjugating peptides with nanoparticles, it is possible to improve the stability, solubility, and controlled release of the peptides, enabling more efficient targeting of specific cells or tissues. This synergy between peptides and nanoparticles also opens doors for enhancing the therapeutic efficacy of peptide-based drugs while minimizing potential side effects 148, 149. Additionally, nanoparticle-mediated delivery systems can facilitate peptide penetration through biological barriers, such as the blood-brain barrier, thus expanding the scope of peptide therapies. The incorporation of peptides with nanoparticles also holds promise in diagnostic applications, such as imaging and biosensing, by leveraging the unique optical, magnetic, or surface plasmon resonance properties of the nanoparticles 148, 150. Overall, the combination of peptides and nanoparticles is poised to revolutionize the fields of targeted therapy, personalized medicine, and diagnostics, offering more precise, effective, and safer treatment options.

Recent studies have explored the potential of peptide-modified nanoparticles for enhanced wound healing and skin tissue regeneration. Among these, myristoyl tetrapeptide-6-silver nanoparticles (MT6-AgNPs) and GHK-Cu-silver nanoparticles (GHK-Cu-AgNPs) have exhibited enhanced wound healing capacity compared to free peptides or unmodified AgNPs 151. This study highlights an innovative approach to wound healing by combining the bioactive properties of peptides with the therapeutic advantages of AgNPs. Both MT-6 and GHK-Cu are known for their roles in enhancing tissue repair and regeneration, but their clinical application has been limited by issues such as low stability, rapid degradation, and poor penetration into target tissues. On the other hand, AgNPs are widely recognized for their antimicrobial properties, which help prevent infections in wounds and accelerate the healing process. MT-6 is a short lipidated peptide that has been shown to promote cell migration and proliferation during wound recovery. The lipidation of the peptide increases its hydrophobicity, enabling better cellular uptake and retention at the site of injury. Similarly, GHK-Cu stimulates collagen synthesis, enhances tissue remodeling, and promotes angiogenesis. GHK-Cu possesses antioxidant properties, which can help to reduce oxidative stress and inflammation in the wound area, further supporting wound recovery. In addition, the antioxidant biomaterials also play a crucial role in cutaneous wound healing and tissue regeneration by mitigating oxidative stress, reducing inflammation, and promoting cellular proliferation and ECM remodeling, ultimately accelerating the repair process 65, 151, 152. Within this context, GHK-AgNP conjugates have emerged as a multifunctional nanoplatform that integrates antimicrobial protection with enhanced tissue regeneration 36.

GHK-AgNPs, previously described for their combined regenerative and antimicrobial effects, can be better appreciated when placed in the broader context of other peptide-nanoconjugate platforms 36. For instance, RGD-functionalized nanoparticles are widely studied for targeted delivery, utilizing the RGD motif's high affinity for integrin receptors overexpressed in angiogenic endothelial cells. These systems have shown enhanced cell adhesion, migration, and tissue regeneration in wound healing and cancer therapy models 153. Similarly, LL-37-modified nanoparticles have demonstrated improvements in collagen synthesis, antimicrobial activity, and modulation of inflammatory responses 154. Compared with these systems, GHK-AgNPs uniquely combine the broad-spectrum antimicrobial properties of silver nanoparticles with GHK's strong regenerative capacity, including stimulation of collagen deposition, angiogenesis, and antioxidant activity. This dual functionality positions GHK-AgNPs as a multifunctional therapeutic platform that can address both microbial control and tissue repair, offering a distinct advantage over single-function peptide-nanoconjugates 36.

### 4.4 Synergistic Effects with the Combination of Other Bioactive Peptides

Expansive research efforts have been carried out focusing on peptide drug discovery, drug design, peptide synthesis, structural modification, activity evaluation, and toxicity risks of peptide drugs 15, 155, 156. Current technological advancements, including nanomedicine, have further propelled the research on novel applications of therapeutic peptides, in which several carriers for drug delivery systems that entrap and deliver peptides to the target sites have been suggested. The synergistic effects of tripeptides when combined with other peptide classes, such as tetrapeptides and hexapeptides, have also been the subject of recent research.

In brief, TriHex^TM^ (ALASTIN Skincare, Inc., Carlsbad, CA) discoveries present a breakthrough in wound healing and skin regeneration. A combination of tripeptide and hexapeptide (TriHex) is specifically engineered to restore skin condition by eliminating clamped collagen and elastin fragments while promoting the synthesis of new collagen and elastin. TriHex^TM^ also confers additional benefits in wound healing 61, 62, 89. A trial by Wilson et al. 89 reported better healing and improved patient symptomology, less erythema, exudation, tenderness, burning/ stinging, and better skin quality in the TriHex group compared to the standard of care. The tripeptide/ hexapeptide system potentially induces a shift from an inflamed environment to a pro-regenerative environment through the promotion of neocollagen and elastin synthesis, thus optimizing ECM, contributing to faster healing 89. Similarly, a study by Widgerow et al. 62 demonstrated skin recycling mechanisms by the TriHex system via the removal of aged, damaged skin fragments and the restoration of an optimal environment with increased new collagen and elastin. ECM modulation through significant ECM improvement was observed, including better distribution of new, healthy collagen in the entire ECM frame of the tested skin region in the TriHex-treated group compared to the apparent distribution of mature, older collagen in the ECM baseline in the non-treated group 62. Recently, TriHex 2.0 has been adapted, where an octapeptide (consisting of eight amino acids) is incorporated into the tripeptide-hexapeptide system. Compared to the original TriHex, the new approach exhibited an enhanced wound-healing capacity. Gene upregulations, including those linked to TGF, can be caused by the synergistic effects of octapeptides with tripeptides and hexapeptides. Through the inhibition of unhealthy collagen and the activation of fibroblasts during wound healing, upregulation of the TGF-β3 pathway may promote scarless wound healing 61.

### 4.5 Self-Assembled Tripeptide Nanoparticles

The versatility of peptides allows for precise control over their properties, including mechanical strength, biodegradability, and biocompatibility, which are critical for creating suitable scaffolds for tissue regeneration. Tailoring the amino acid sequence within self-assembled tripeptides enables the modulation of biological cues such as cell adhesion, growth factor presentation, and enzymatic degradation, promoting cell migration, proliferation, and differentiation. This makes self-assembled tripeptides a promising material for supporting tissue repair and regeneration, from wound healing to more complex tissues such as cartilage, bone, or neural tissues 133, 157-160. For example, Chen et al 75 recently developed a self-healing hydrogel (EW/OKGM@GHK-Cu, GEK) by combining oxidized konjac glucomannan (OKGM) with fresh egg white (EW). The material was cross-linked via a Schiff base and further functionalized with the bioactive tripeptide GHK-Cu. The GEK hydrogel exhibited antibacterial and anti-inflammatory activity, promoted tissue adhesion and neovascularization, and supported skin regeneration, highlighting its potential as a biocompatible and effective dressing for infected wound healing 75.

## 5. Other Applications of Tripeptides

### 5.1 Cosmetic Applications

Globally, the cosmeceutical industry continues to grow rapidly, commercializing products with a variety of functions for the skin. Aimed primarily to improve the skin appearance with additional benefits using the topical application of formulations with bioactive ingredients, extensive efforts have been propelled along with technological advancements in recent decades. Bioactive peptides have garnered significant interest from the cosmetics sector due to their potential. Cosmetic peptides generally help increase the viability and proliferation of skin cells, decrease skin pigmentation, reduce tissue inflammation, and improve skin barrier function 161. The tripeptide GHK-Cu was discovered in 1973 and has since become a popular ingredient in skin treatments due to its potent protective and regenerative properties 23. In September 2023, a submission concerning the topical cosmetic application of GHK-Cu, accompanied by supporting documentation, was made to regulatory authorities. To date, GHK-Cu continues to be widely commercialized due to its abundant skin benefits 23, 162. GHK-Cu can penetrate the stratum corneum, where it helps reverse skin thinning, tighten loose tissue, repair the barrier, strengthen the dermal structure, reduce wrinkle depth and fine lines, and improve clarity, elasticity, and suppleness. Likewise, GHK-Cu has been shown to smooth rough skin, reduce lesions and spots, alleviate hyperpigmentation and photodamage, enhance overall skin appearance, accelerate wound healing, protect against ultraviolet radiation, and mitigate inflammation and free radical-induced damage 23. Several studies confirmed the cosmeceutical benefits of this tripeptide. Badenhorst et al. 59 observed that the human adult dermal fibroblasts incubated with GHK-Cu at 0.01, 1, and 100 nM resulted in enhanced collagen and elastin synthesis. Dymek et al. 69 delineated the conjugation of GHK-Cu with liposomes as a carrier system in causing elastase inhibition, which lowers the rate of elastin degradation and maintains the skin's structural integrity.

#### 5.1.1 Scarless and Scar-Minimizing Wound Healing

Recent research highlights tripeptides as promising agents for scar-minimizing and potentially scarless wound healing, owing to their ability to modulate ECM remodeling, regulate fibroblast activity, and influence growth factor signaling such as the TGF-β3 pathway. GHK-Cu, the most studied tripeptide in this context, has been shown to promote balanced collagen synthesis, accelerate epithelialization, and reduce fibrosis in both *in vitro* and preclinical models 70. Formulation innovations, such as the TriHex peptide system, which combines tripeptides with hexapeptides, and more recently octapeptides in the TriHex 2.0 adaptation, have demonstrated synergistic upregulation of regenerative genes and suppression of aberrant collagen deposition, a process closely linked to reduced scarring. These strategies build on earlier cosmetic applications, where preoperative skin conditioning and ECM clearance improved dermal receptivity for regeneration 62, 89. However, most mechanistic insights derive from studies involving multi-peptide formulations or larger peptides, with limited tripeptide-only clinical evidence 163, 164. Addressing this gap will be essential to establish tripeptides as a standalone therapeutic class in scarless wound healing.

### 5.2 Treating Bacterial Infections in Wounds

#### 5.2.1 Role of Tripeptides in Combating Bacterial Infections in Wounds

Wounds and microbial infections are closely associated, as open wounds provide a passage of entry for pathogens, typically bacteria, to enter the skin and multiply, subsequently causing infections. These infections may trigger pathological mechanisms leading to multiple complications, such as delayed wound healing and persistent infections 165. Commonly, bacteria, including *S. aureus*, *P. aeruginosa*, and *E. coli* are found to colonize wounds, causing infection in the wound area, as well as producing biofilms that may further exacerbate the infection. Conventionally, antibiotics are often resorted to as treatments for bacterial infection in wounds. However, antimicrobial resistance continues to pose a major challenge worldwide. Each year, an estimated 1.27 million deaths are attributed to bacterial infections caused by pathogens resistant to existing antibiotics 166. Host defense peptides, also known as AMPs, were first discovered in the 1980s. AMPs demonstrate their strong and wide-ranging antimicrobial activity against viruses, fungi, and bacteria. Studies associating tripeptides with antimicrobial properties have been actively conducted, suggesting potent antimicrobial activities in specific tripeptides 167. In a study, GHK-Cu-AgNPs demonstrated the strongest antibacterial effects against *S. aureus*, while GHK-AgNPs exhibited the strongest antibacterial activity against *E. coli*. The minimum inhibitory concentration (MIC) against *S. aureus* and *E. coli* was the lowest for both GHK-AgNPs and GHK-Cu-AgNPs (MIC: 8 µg/ml). Similarly, GHK-Cu-AgNPs exhibited the strongest inhibitory effect in mice models infected with *S. aureus*, according to *in vivo* antibacterial investigations 36. In another study investigating the Fmoc-Tripeptide hydrogel coating, self-assembled Fmoc-FFY (Fmoc: 9-fluorenylmethoxycarbonyl; F: phenylalanine; Y: tyrosine) displayed greater antibacterial activity than Fmoc-FFpY (p: PO_4_^2-^) peptide in solution at low concentrations (≤0.5 µg/mL). After 24 hours, the development of the Gram-positive* S. aureus* was completely inhibited by Fmoc-FFY hydrogel coatings 157. More robust evidence is needed to support the potential of specific tripeptides as emerging alternatives to conventional antibiotics, providing a viable approach to address drug-resistant pathogens.

#### 5.2.2 Potential Use in Treating Resistant Infections and Biofilm-Associated Wounds

The role of AMPs in the combat against drug-resistant bacterial infections has been extensively studied. Previously, Shao et al. 58 revealed antibacterial properties possessed by *in situ* mucoadhesive hydrogel entrapping tripeptide KPV in gingival ulcer wounds infected with MRSA. In another study, tripeptide thiol-antioxidant glutathione exhibited antibacterial activity against the tested isolates of carbapenem-associated multidrug resistance. Besides, the synergistic mechanism between the tripeptide and carbapenem (meropenem) was indicated by the consistent record of significant fractional inhibitory concentration (FIC) (*p* < 0.5). Furthermore, the carbapenem-associated multidrug-resistant isolates were killed by subinhibitory concentrations of both glutathione and meropenem at > 2log_10_ within 12 hours 168. The formation of biofilms during chronic bacterial infection can induce biofilm-associated wounds. In a study by Chingizova et al. 169, asterripeptides A-C, which are marine fungal tripeptides that include a cinnamic acid moiety, were investigated for their wound healing capacity in *S. aureus* ATCC 21027 strain-infected skin. Biofilm formation was significantly inhibited by approximately 63% following the treatment with 10 µM of asterripeptides A-C. In another study, the novel small lipotripeptides DICAMs, which are non-cationic fatty amine-tripeptide conjugates, effectively prevented the formation of biofilms by breaking down biofilms and eliminating the biofilm-affixed bacteria 91.

### 5.3 Cancer Therapy

Emerging research involving tripeptides as anticancer agents has explored alternative strategies to improve survival rates in cancer patients worldwide. Computational studies suggest that certain tripeptides may function as vehicles for drug delivery systems. For example, the tripeptide RGD, a common cell-targeting peptide, can serve as a targeting moiety for the anticancer drug cyclophosphamide (CP), offering high loading capacity and the potential for slow, prolonged drug release. RGD selectively binds to integrins, which are cell surface receptors involved in cell-cell and cell-ECM interactions, and are implicated in promoting cancer cell migration and proliferation. Increased RGD uptake by tumor cells could potentially occur due to the binding affinity of RGD to integrins. Therefore, conjugating CP to RGD's active sites may facilitate targeted delivery and enhance interference with tumor cell growth, although further experimental validation is needed to confirm these effects 170.

GHK can be utilized as a complementary cancer therapy due to its potent anti-cancer properties. Park et al. 60 studied the effects of GHK-Cu both *in vitro* (lipopolysaccharide-induced RAW 264.7 macro-phages) and *in vivo* (acute lung injury in mice). In their study, it was suggested that GHK-Cu possesses protective effects in lipopolysaccharide-induced acute lung injury. This result also implies the application of GHK-Cu in cancer therapy. By blocking the activation of certain proteins, GHK-Cu inhibits specific signaling pathways responsible for cell proliferation, survival, angiogenesis, and invasion. This disruption helps prevent cancer formation and potentially supports targeted cancer therapies. In both *in vitro* and *in vivo* models, the study reported that GHK-Cu reduced the production of TNF-α and IL-6 through the suppression of NFκB p65 and p38 mitogen-activated protein kinase (MAPK) signaling. Expressions of TNF-α, IL-6, NFκB's p65, and p38 are frequently upregulated in cancer and have been associated with tumor progression. Activation of p38 MAPK signaling contributes to the regulation of cancer stem cell (CSC) signaling pathways in multiple cancer types. CSCs are a subpopulation of tumor cells with the capability to self-renew and differentiate, which drives tumor progression and metastasis. CSCs have been linked to chemoresistance in cancer patients, which accounts for 90% of treatment failures. GHK-Cu's attenuation of p38 MAPK signaling offers a new paradigm to combat chemoresistance in cancer patients. Further, the study demonstrated reduced levels of ROS and enhanced activities of superoxide dismutase, which have been linked to cellular survival, leading to many pathological conditions such as cancer. This result suggests GHK-Cu's effects on mitigating the deleterious effects of ROS 23, 60, 171, 172.

### 5.4 Use of Tripeptides for Neuroprotection

GHK promotes neurological recovery through the stimulation of nerve outgrowth, restoration of skin innervation via the increase in the synthesis of neurotrophic factors, alleviation of neuronal apoptosis, and decline in neurological deficits following injury 23, 173. In a study, it was found that GHK stimulated nerve outgrowth in severed rat nerves in a collagen tube infused with the tripeptide. Meanwhile, in comparison to the control group, GHK-Cu accelerated the regeneration of nerve fibers from nerve stubs placed in a collagen tube, increased the number of axons and the proliferation of Schwann cells, and boosted the synthesis of nerve growth factor and the neurotrophins NT-3 and NT-4 23. In another study, compared to the control group, GHK treatment groups (1 or 10 mg/kg) demonstrated decreased neuronal apoptosis and neurological deficits in rat brains induced by intracerebral hemorrhage (ICH) injury. GHK showed a notable improvement in neurological deficit scores and a marked rise in the number of intact neurons three days after collagenase VII was administered. Furthermore, as demonstrated by the findings of the TUNEL analysis, GHK administration resulted in a notable decrease in apoptotic cells, suggesting that neuronal death was inhibited, accelerating neurological recovery 173. Neural regeneration following an injury can be potentially treated with Gly-Pro-Glu (GPE), the amino-terminal peptide of the insulin-like growth factor-1 (IGF-I).

**Figure 6:**
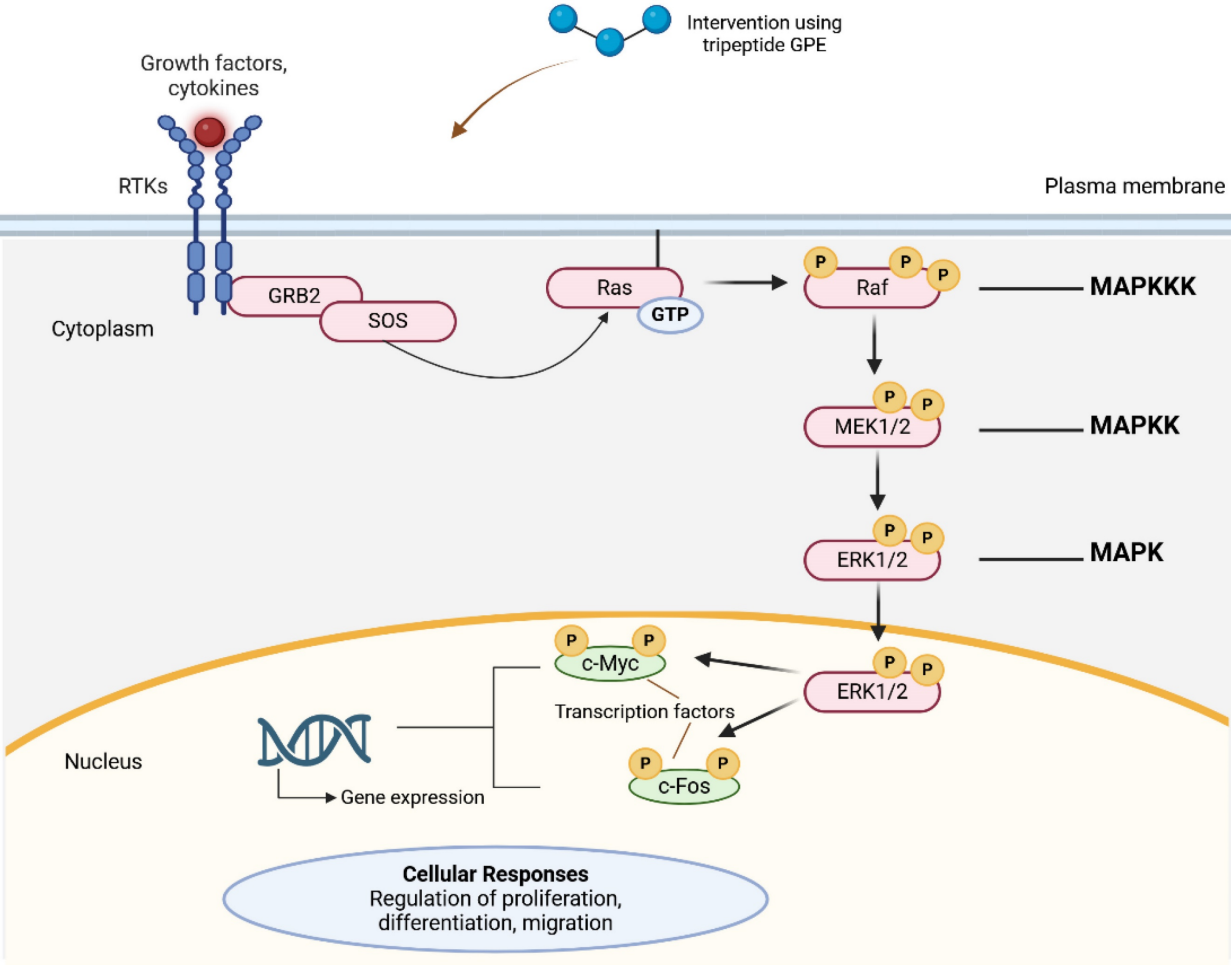
** Overview of the effect of tripeptide GPE on the ERK signaling pathway.** Growth factors such as (e.g., VEGF, platelet-derived growth factor [PDGF] bind to a receptor tyrosine kinase (RTK) on the cell membrane, causing autophosphorylation of the receptor, thus creating docking sites for intracellular signaling proteins. Growth factor receptor-bound protein 2 (GRB2), an adaptor protein, binds to the receptor and recruits Son of Sevenless (SOS), a guanine nucleotide exchange factor (GEF). SOS activates Ras, a small guanosine triphosphatase (GTPase), by exchanging guanosine diphosphate (GDP) for guanosine triphosphate (GTP). This triggers the MAPK cascade, such that Ras-GTP activates Raf [also called MAPK kinase kinase (MAPKKK)], which in turn activates MEK1/2 [MAPK kinase (MAPKK)]. Activated MEK1/2 then activates ERK1/2 (MAPK). Once activated, ERK1/2 is transferred to the nucleus, where it phosphorylates transcription factors such as c-Myc and c-Fos, leading to the expression of genes responsible for cellular regulations such as cell proliferation, differentiation, and migration. Illustration created with BioRender.com.

**Figure 7:**
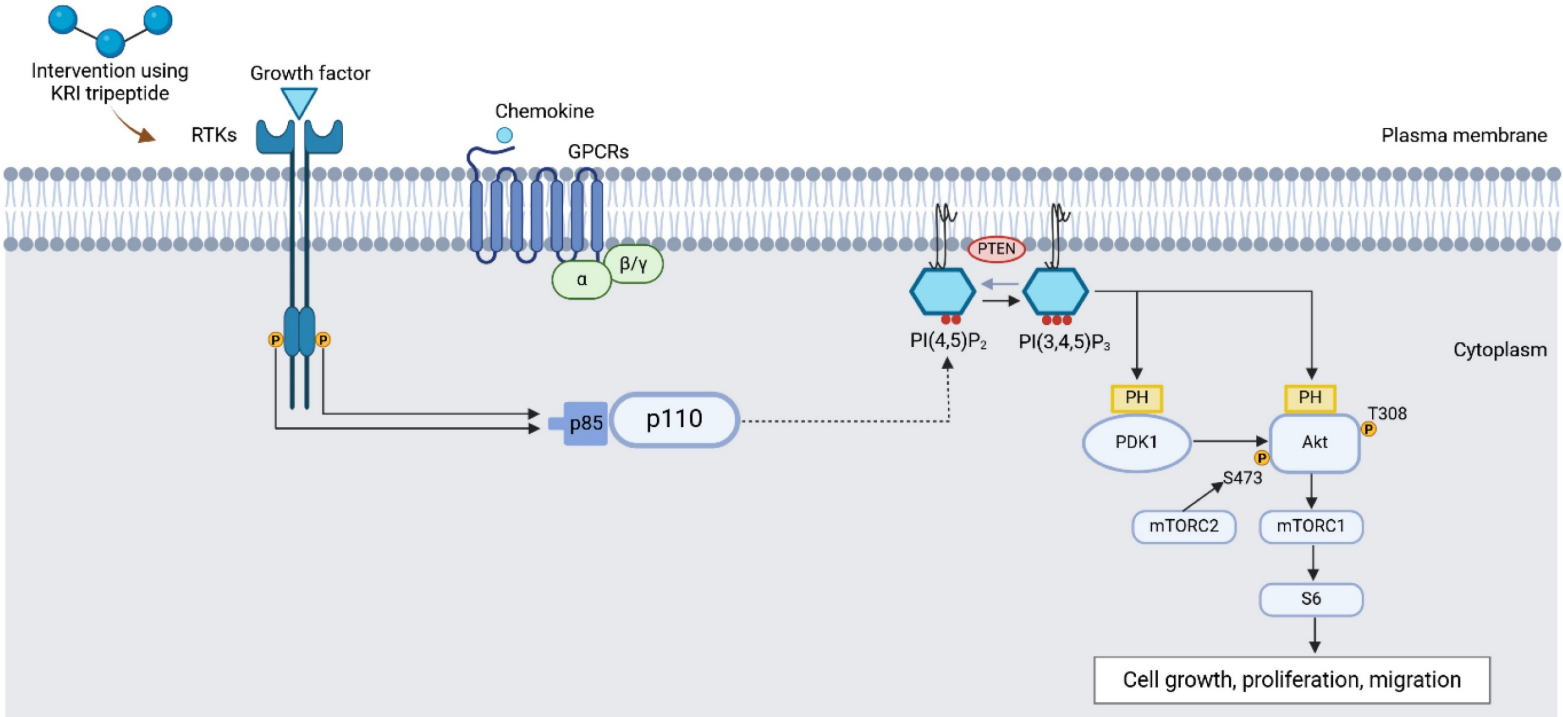
** Overview of the effect of tripeptide GPE on the PI3K/Akt signaling pathway.** Ligand-induced phosphorylation of the RTKs induces recruitment of the regulatory subunit of PI3K (p85) to the RTKs, relieving the inhibitory interaction with the catalytic subunit (p110) and resulting in activation. The G-protein beta and gamma subunits (Gβγ) subunits downstream of G-protein-coupled receptors (GPCRs), also trigger activation of p110. Activated PI3K enzymes are brought to the membrane, where phosphatidylinositol 4,5-bisphosphate (PIP_2_) is converted into phosphatidylinositol 3,4,5-trisphosphate (PIP_3_). Production of PIP_3_ enables phosphorylation of Akt at threonine 308 (T308) by 3-phosphoinositide-dependent protein kinase-1 (PDK1) and at serine 473 (S473) by the mechanistic target of rapamycin complex 2 (mTORC2). PIP_3_ also interacts with proteins possessing a pleckstrin homology (PH) domain, such as protein kinases, including Akt and Bruton's tyrosine kinase (BTK). Activation of downstream effectors, including the mechanistic target of rapamycin complex 1 (mTORC1) and ribosomal protein S6 (S6), regulates protein synthesis, cell growth, and metabolism. Phosphatase and tensin homolog (PTEN) dephosphorylates PIP_3_ back to PIP_2_, thereby regulating the pathway. Illustration created with BioRender.com.

A study by Almengló et al. 24 revealed neuroprotection and neuroregeneration by GPE in which the tripeptide enhanced proliferation and migration in mouse neural stem cells (NSCs) following injury involving 13.5 Days post-conception (dpc) mouse embryos. The impact of GPE on cell proliferation, migration, and survival was examined under basal conditions and in response to a wound-healing assay, relative to two other treatment groups: growth hormone (GH) and GPE + GH groups. Compared to the vehicle and GPE + GH groups, GPE effects in NSCs proliferation were shown by a significant increase in BrdU+ cells (representing labelled proliferating cells), while significant migration by GPEs in NSCs was presented by the wound closure rate, which was independent of cell proliferation. Cell proliferation induced by GPE was further delineated by an increased level of phosphorylated Akt, indicating potential upregulation of the ERK (**Figure 6**) and PI3K/Akt signaling pathways (**Figure 7**) pathways, which are among the signaling pathways involved in the regulation of cell proliferation. Interestingly, GH demonstrated a relatively similar rate of cell proliferation and migration, thus suggesting the potential application of GH for neuroprotection and/ or neuroregeneration 24.

### 5.5 Cardiovascular Diseases

High blood pressure (hypertension) is usually regulated by angiotensin-converting enzyme (ACE) via the kinin-kallikrein system (KKS) and the renin-angiotensin-aldosterone system (RAAS). Through the cleavage of the C-terminal dipeptide, ACE alters the molecular structure of bradykinin in KKS, causing a loss of function that results in vasoconstriction and elevated blood pressure. Meanwhile, in RAAS, ACE's conversion of angiotensin I to angiotensin II, which has a potent vasoconstrictor impact, may cause the adrenal cortex to generate more aldosterone, both of which raise blood pressure. ACE inhibitors (ACEIs) (e.g., first-line antihypertensive drugs, such as lisinopril, enalapril, captopril) are among the most commonly prescribed antihypertensive drugs and are widely used in the treatment of cardiovascular disorders 66. ACE-inhibitory peptides potentially block the active site of ACE and diminish the conversion of angiotensin I to angiotensin II, thus alleviating blood pressure 117. Jujube 174, earthworm protein 175, and soy protein 176 are among the natural plant and animal protein sources from which ACE-inhibitory peptides have been successfully extracted and discovered by several studies in recent years. In a study, it was found that a significant ACE inhibition was induced by casein-derived ACE-inhibitory tripeptide Leu-Leu-Tyr (LLY) through a conformational shift in ACE. LLY showed potent ACE inhibition (IC₅₀ = 44.16 ± 2.45 μM) and remained stable across varied pH, moderate heat, metal ions, glucose, and salt. It acted via non-competitive inhibition, binding to non-active sites and inducing structural rearrangements, with reduced α-helical content and increased β-sheet and random coil, thereby disrupting ACE function without active-site competition. This potency, stability, and distinct mechanism support the potential of ACE-inhibitory tripeptides for functional foods or complementary hypertension therapies 177.

In spontaneously hypertensive rats (SHRs), the tripeptides Thea-Thea-Pro (TTP) and GABA-GABA-Pro (gAgAP) produced a sharp and rapid reduction in systolic blood pressure (SBP). TTP treatment lowered SBP to a similar extent as the clinical standard captopril, although the duration of the antihypertensive effect was shorter in the TTP group. Both peptides demonstrated strong ACE-inhibitory activity, with IC₅₀ values of 0.92 μmol/L for TTP and 3.4 μmol/L for gAgAP. In addition, both TTP and gAgAP downregulated transcription levels of *angiotensin II receptor type 1* (*agtr1*) and miR-132/-212, with TTP producing a comparable reduction in *agtr1* to captopril and a significant decrease in miR-132/-212 expression. These findings indicate that TTP, in particular, may serve as a potential antihypertensive candidate with potent ACE-inhibitory activity 177. Nevertheless, despite demonstrating potent *in vivo* effects, neither study performed side-by-side dose-response benchmarking against clinical ACE inhibitors (e.g., lisinopril or captopril) under identical assay conditions. This omission limits pharmacological context, hinders potency normalization across studies, and constrains the prioritization of peptide leads for translational development 177, 178.

In addition to antihypertensive activity, tripeptides such as GHK-Cu have been reported to contribute to vascular repair following injury. GHK-Cu restores blood flow into damaged tissues through a combination of angiogenesis, anticoagulation, and vasodilation, partly by upregulating bFGF and VEGF. Moreover, GHK-Cu promotes the synthesis of collagen and elastin, thereby reinforcing the structural integrity of vessel walls. Importantly, proteolytic cleavage of SPARC (secreted protein acidic and rich in cysteine), a matricellular protein abundantly expressed at sites of vascular injury and tissue remodeling, generates GHK-containing fragments that promote endothelial cell proliferation and neovascularization. Once blood flow is re-established, intact SPARC restrains further angiogenesis, providing a built-in mechanism of regulation. These findings position GHK-Cu as a promising vascular-healing tripeptide with potential relevance for cardiovascular disease beyond blood pressure regulation 23.

Collagen-derived tripeptides (CTP) have demonstrated vascular-protective properties in preclinical models and early human investigations, where they enhanced endothelial resilience under oxidative stress and attenuated vascular inflammation. These findings point to a potential role for CTP in stabilizing atherosclerotic plaques and mitigating vascular dysfunction, although the underlying molecular mechanisms remain to be fully elucidated 179, 180. Beyond naturally occurring sequences, rationally designed tripeptides have also emerged as promising candidates; for instance, DT-109 (Gly-Gly-Leu) markedly reduced atherosclerosis and vascular calcification in nonhuman primates, underscoring the translational potential of tripeptide-based therapeutics for cardiovascular diseases 181. Together, these findings highlight that while ACE-inhibitory peptides remain the most extensively studied, peptide-based strategies in cardiovascular diseases potentially extend beyond blood pressure regulation to include vascular repair, plaque stabilization, and endothelial modulation.

### 5.6 Food and Nutritional Supplements

#### 5.6.1 Tripeptides as Functional Food Components for Health Promotion and Disease Prevention

The gut hormones glucagon-like peptide 1 (GLP-1), oxyntomodulin (OXM), and peptide tyrosine tyrosine (PYY) are elevated following gastric bypass surgery. The tripeptide gut hormone infusion GOP, which stands for GLP-1, OXM, and PYY, was investigated in a trial by Behary et al. 182 involving obese and pre-diabetic/ diabetic patients for its potential dietary and therapeutic benefits. Compared to the placebo, the tripeptide infusion reduced mean daily food intake by 27%, decreased fat intake, increased protein intake, and enhanced satiety, while not altering food preferences and sweet taste function. However, GOP may promote restrained eating, which is associated with alleviated activation of the brain reward area. Collectively, these effects may contribute to weight loss post-surgery in obese patients, suggesting the functional use of tripeptides for weight loss 182.

CTP, which is rich in Gly-X-Y motifs, can be incorporated into functional foods as a preventive strategy for vascular health. In a study by Tomosugi et al. 179, daily CTP supplementation in healthy adults improved lipid metabolism and vascular indices associated with atherosclerosis risk. After six months, high-density lipoprotein cholesterol (HDL-C) and triglyceride (TG) levels were significantly increased in the low-risk group, while the low-density lipoprotein cholesterol (LDL-C)/HDL-C ratio decreased in the high-risk group, suggesting favorable lipid modulation. In addition, serum toxic advanced glycation end-products (TAGE), biomarkers of vascular wall damage, were markedly reduced across all participants. The cardio-ankle vascular index (CAVI), a prognostic marker for cardiovascular events, declined by approximately 0.2 points bilaterally, reflecting improved arterial elasticity. These findings highlight CTP's potential as a functional food component that supports cardiovascular resilience, particularly in individuals at elevated risk of atherosclerosis 179, 183-185.

#### 5.6.2 Role in Muscle Repair and Recovery in Sports Nutrition

Protein hydrolysate (PH) allows faster absorption of amino acids by the body than intact proteins, thus maximizing the transport of nutrients to muscle tissues, making it a unique application in sports medicine. Enriched in di- and tripeptides, PH facilitates protein synthesis of the skeletal muscle via anabolism, leading to an increase in skeletal muscle mass 186, 187. Consequently, PH will result in swifter muscle recovery and remodeling following exercise/ muscle injury 186, 188. In a study by Brown et al. 188, whey PH assisted muscle recovery and alleviated muscle damage in exercise-induced skeletal muscle damage (EIMD) as evidenced by decreased CK levels (an EIMD biomarker) and reduction of muscle function loss. Additionally, PH may confer potent insulinotropic effects, allowing the permissive effects of insulin on muscle protein synthesis (MPS) 186. A study involving a 2-hour swimming protocol in rats revealed that whey HP ingestion induced significantly higher MPS compared to the intact whey protein 189. Albeit requiring more robust evidence and elucidative mechanisms, the specific tripeptides in PH, along with particular dipeptides, are potentially efficacious in contributing to muscle repair and recovery.

## 6. Future Prospects and Challenges in Tripeptide Research

The prospects and challenges in tripeptide research are shaped by the growing understanding of the complex biological roles and potential applications of tripeptides in medicine, nutrition, and biotechnology. The recent advancement in tissue engineering and regenerative medicine (TERM) has shed light on the application of peptide modification, which potentially results in enhanced medication delivery, reduced harm to healthy tissues, and enhanced cell targeting. The innovation of self-assembled peptides has been proven to demonstrate high biocompatibility and biological activities, making them attractive as therapeutic drugs 160. There is increasing interest in developing tripeptides with targeted therapeutic functions, particularly for applications in wound healing 36, 75, 92, as well as in cancer 60, 170, neurodegenerative 24, 173, and cardiovascular diseases 177, 178, and in the design of functional foods 179, 182, 188. However, significant challenges remain, including the synthesis of stable tripeptides with good tissue penetration, insufficient understanding of their mechanisms of action, the scarcity of studies in larger animal models and human trials, and the lack of direct head-to-head comparisons with larger peptides 75, 95. Many studies also rely on small sample sizes, underscoring the need for larger, more diverse populations to validate efficacy, safety, and patient compliance. Moreover, peptides are sensitive to chemical and physical instability, increasing the risk of degradation during development and storage. While alternative administration routes, such as intravenous, intramuscular, nasal, percutaneous, and transdermal delivery, have shown promise in certain conditions, these methods can be invasive and uncomfortable for patients. Therefore, extensive future research is needed to address these issues and improve the delivery and patient experience 151, 190.

In addition to stability and delivery challenges, peptides, particularly those derived from non-human sources, carry the risk of triggering immune responses. This can lead to the development of specific antibodies that neutralize the drug or cause allergic reactions, potentially resulting in serious complications for patients. To mitigate these risks, comprehensive *in vitro* and *in vivo* studies evaluating the immunogenic potential of peptide compounds should be conducted before initiating clinical trials. Additionally, certain peptide sequences may be recognized as foreign by the immune system, increasing the likelihood of unwanted immune reactions. This risk is further compounded by the presence of impurities in synthetic peptides 191. Tripeptides such as GHK-Cu have an established safety record in topical and cosmetic applications, showing low cytotoxicity and reduced skin irritation compared with other copper compounds, and acceptable tolerability in clinical cosmetic studies 23, 74, 93. However, dedicated immunogenicity data are sparse; formal analyses of antibody formation or systemic immune responses after topical or parenteral administration are lacking. Therefore, while existing evidence supports low local toxicity, comprehensive *in vitro* and *in vivo* immunogenicity testing remains advisable prior to clinical translation 71, 191. Additionally, the use of tripeptides derived from animal sources, such as bovine and porcine, may raise social, religious, and cultural concerns. To address these issues, drug developers should consider alternative sources, such as jellyfish, fish, or sponges, for peptide extraction. By exploring these options, developers can mitigate potential objections and ensure broader acceptance of peptide-based therapies 192.

## 7. Conclusion

Beyond wound healing, tripeptides exhibit a broad range of biological activities. They have been shown to possess important roles in wound healing, skin regeneration, antimicrobial activities, cancer cell cytotoxicity, neurological and cardiovascular recovery and protection, cosmeceutical, food and dietary supplementation, muscle repair, and sports nutrition. Their ability to mimic or influence the action of larger bioactive molecules also makes them promising candidates for drug development and therapeutic applications. Furthermore, their small size allows for easy synthesis and modification, facilitating their incorporation into drug-delivery systems or peptide-based therapeutics. Recent technology, such as nanomedicine, further enhances tripeptide effects in many therapies. While the potential of tripeptides in biomedical applications has been proven significant, challenges such as stability, bioavailability, and immunogenicity need to be addressed. However, ongoing advancements in peptide chemistry and biotechnology are expected to overcome these barriers, expanding the therapeutic use of tripeptides. Their versatility in promoting healing, modulating biological processes, and serving as drug candidates positions them as essential tools in modern medicine, offering hope for more efficient treatments for a variety of conditions, including wounds, infections, and chronic diseases.

## Figures and Tables

**Table 1:** Comparative Advantages and Limitations of Tripeptides and Larger Peptides in Biomedical Applications.

Criteria	Peptide Types	
	Tripeptides (e.g., GHK, GHK-Cu, Pal-GHK)	Larger Peptides (e.g., CyRL-QN15, LL-37, AW1)
Size & Structure	3 amino acids, linear 69	>3 amino acids, cyclic 25, 67, 68
Advantages	Low-cost synthesis 23, 29, 70 Low immunogenicity 29, 71 Easily synthesized using standard methods such as solid-phase peptide synthesis (SPPS) and liquid-phase peptide synthesis (LPPS) 72, 73 Easy to modify chemically 29, 72 Low toxicity risks 23, 74 High biocompatibility 71, 75	Offer more diverse functions and potential benefits 76 Generally more stable in a chronic wound environment due to longer sequence, but still prone to protease degradation 67, 77 Known potent antimicrobial activity (e.g., LL-37, AW1) 25, 78
Limitations	Poor *in vivo* stability due to its susceptibility to degradation by skin proteases; can be stabilized by conjugation with biotin (Bio-GHK) or with palmitic acid (e.g., Pal-GHK) 71, 79 Poor penetrability across the skin due to its hydrophilic nature; can be enhanced using carriers such as liposomes or nanoliposomes, or conjugated with palmitic acid (e.g., Pal-GHK) 23, 71 Lack of clinical trial data (e.g., GHK-Cu and Pal-GHK) 80	Limited tissue penetration (e.g., LL-37) 81 Increased susceptibility to enzymatic degradation (e.g., LL-37) 81, 82 Complex synthesis and high production cost (e.g., LL-37) 81, 82 Cytotoxicity risks (e.g., LL-37) 81, 83 Lower activity in physiological environments (e.g., LL-37) 81

**Table 2:** Comparative Analysis between GHK Tripeptides with Larger Peptides in Wound Healing Models.

Peptide	Wound Models Used	Main Mechanisms	Key Outcomes in Wound Healing	Refs.
GHK (Tripeptide)	Mostly *in vivo* rodent wounds, some *in vitro* fibroblast/keratinocyte studies	Copper chelation, stimulation of ECM protein synthesis (collagen, elastin, glycosaminoglycans [GAGs]), angiogenesis promotion	Accelerated epithelialization Improved collagen remodeling Enhanced angiogenesis	23, 36, 85
CyRL-QN15 (7 aa)	*In vivo* full-thickness mouse wounds (normal + diabetic); *in vitro* keratinocytes	Downregulation of miR-365-2-5p leads to increased expression of sirtuin 1 (SIRT1) and Forkhead box protein O1 (FOXO1), decreased signal transducer and activator of transcription 2 (STAT2), reduced inflammation, and activation of the Wnt/β-catenin pathway in hair follicles	Promoted keratinocyte proliferation/ migration Reduced inflammation Stimulated hair follicle regeneration	86, 87
LL-37 (37 aa)	Methicillin-resistant Staphylococcus aureus (MRSA)-infected mouse wounds (full-thickness)	Antimicrobial (membrane disruption), immunomodulatory, vascular endothelial growth factor (VEGF) upregulation	Reduced bacterial load Enhanced re-epithelialization, granulation, collagen organization, angiogenesis	88
AW1 (72 aa)	Diabetic and burn mouse wounds; *in vitro* macrophages & endothelial cells	Binding to Toll-like receptor 4 (TLR4) activates nuclear factor-kappa B (NF-κB) signaling, leading to macrophage polarization and pro-angiogenic effects.	Reduced inflammation Improved re-epithelialization, angiogenesis, granulation tissue formation	25

**Table 3:** Effects of tripeptides in wound healing and skin regeneration.

Name of Tripeptide	Tripeptide Sequence/ Modification	Aim	Study Design	Findings	Refs.
Glycine-Histidine-Lysine	Conjugation of Gly-His-Lys (GHK) with silver (Ag) and copper (Cu) has led to the development of GHK-AgNPs and GHK-Cu-AgNPs.	To accelerate cell migration, to promote wound healing, and to elicit antibacterial activities	***In vitro*:** Minimum inhibitory concentration (MIC), minimum bactericidal concentration (MBC), histological analysis, wound scratch assay in mouse dermal fibroblasts (PAM212 and L929) ***In vivo*:** Inoculation of *S. aureus* infection on the wound surface of mouse models	• Increased cell migration • Enhanced dermal fibroblast recovery • Accelerated wound closure • Potent inhibitory activity against *E. coli* and *S. aureus*, inhibitory effect in mice infected with *S. aureus*	36
	Incorporation of Gly-His-Lys (GHK) and α-L-arginine with hydrogel dressing	To stimulate wound healing	***In vivo*:** Skin injury model in rats	• Accelerated cell migration • Faster and better wound recovery • Tissue remodulation occurred	85
TriHex	Conjugation of Gly-His-Lys (GHK) with hexapeptide-12	To restore skin condition	**Clinical trial:** Randomized, single-blinded trial	• Eliminated clamped collagen and elastin fragments • Enhanced new collagen and elastin synthesis • Accelerated wound healing • Improved patient symptomology, better skin quality, less erythema, exudation, tenderness, and burning/ stinging	89
TriHex 2.0	Conjugation of octapeptide with Gly-His-Lys (GHK) and hexapeptide-12	To accelerate wound healing, to promote ECM remodeling	**Ex vivo:** Human discarded facial tissue; elastogenesis assessment, gene expression analysis	• Stimulated elastin synthesis • Enhanced wound healing • Increased collagen production • Activated fibroblasts	61
Lysine-D-Proline-Threonine	Lys-d-Pro-Thr (KdPT)	To mitigate glucotoxicity in diabetes, enhance wound healing, and protect against hyperglycemia	***In vitro*:** Scratch assays on normal human keratinocytes (NHKs), cell viability, metabolic activity and proliferation assays, cell migration assay, and detection of intracellular oxidative stress **Ex vivo:** Human skin organ culture from punch biopsies	• Reduced high glucose (HG)-mediated ROS production in NHKs • Counteraction of the negative effects of hyperglycemic conditions on the viability, proliferation, migration, and metabolic activity of NHKs • Promoted NHK migration, faster wound closure, attenuated HG-mediated suppression of re-epithelialization	90
Lysine-Proline-Valine	Lys-Pro-Val (KPV) incorporated with *in situ* mucoadhesive hydrogels (KPV@PPP_E)	To reduce inflammation, exert antibacterial effects, and promote wound healing	***In vitro***: Anti-inflammatory activities, cell migration assay, and antibacterial activities ***In vivo***: Cytokine analysis, histological analysis in rats with injuries	• Inhibited inflammatory cytokines (IL-1β, TNF-α) • Upregulated anti-inflammatory cytokine IL-10 • Repaired tissue morphology • Antibacterial actions in gingival ulcer wounds infected with MRSA	58
Small lipotripeptides (DICAMs)	Variable sequences	To elicit antibacterial activities	***In vitro*:** Growth-inhibition assays, biofilm assays	• Inhibited biofilm formation • Disintegrated biofilms • Elimination of the bacteria affixed to the biofilm	91
Glycine-Proline-Glutamic acid	Gly-Pro-Glu (GPE)	To provide neuroprotection and regeneration, to accelerate cell proliferation and migration	***In vitro*:** Using mouse embryonic neural stem cells (NSCs) following injury: Cell proliferation assay, scratch wound healing assay, Western blot for phospho-extracellular signal-regulated kinase (ERK) 1/2 and phospho-Akt determination, determination of cell apoptosis	• Neuroprotection and/ or neuroregeneration • Increased proliferation and migration of NSCs • Activated ERK and phosphoinositide 3-kinase (PI3K)/Akt (PI3K/Akt) signaling pathway • Accelerated wound closure	24
